# Mathematical Modeling of Malaria Infection with Innate and Adaptive Immunity in Individuals and Agent-Based Communities

**DOI:** 10.1371/journal.pone.0034040

**Published:** 2012-03-28

**Authors:** David Gurarie, Stephan Karl, Peter A. Zimmerman, Charles H. King, Timothy G. St. Pierre, Timothy M. E. Davis

**Affiliations:** 1 Department of Mathematics, Case Western Reserve University, Cleveland, Ohio, United States of America; 2 School of Physics, The University of Western Australia, Crawley, Western Australia, Australia; 3 The Center for Global Health and Diseases, Case Western Reserve University, Cleveland, Ohio, United States of America; 4 School of Medicine and Pharmacology, The University of Western Australia, Fremantle Hospital, Fremantle, Western Australia, Australia; Institut Pasteur, France

## Abstract

**Background:**

Agent-based modeling of *Plasmodium falciparum* infection offers an attractive alternative to the conventional Ross-Macdonald methodology, as it allows simulation of heterogeneous communities subjected to realistic transmission (inoculation patterns).

**Methodology/Principal Findings:**

We developed a new, agent based model that accounts for the essential in-host processes: parasite replication and its regulation by innate and adaptive immunity. The model also incorporates a simplified version of antigenic variation by *Plasmodium falciparum*. We calibrated the model using data from malaria-therapy (MT) studies, and developed a novel calibration procedure that accounts for a deterministic and a pseudo-random component in the observed parasite density patterns. Using the parasite density patterns of 122 MT patients, we generated a large number of calibrated parameters. The resulting data set served as a basis for constructing and simulating heterogeneous agent-based (AB) communities of MT-like hosts. We conducted several numerical experiments subjecting AB communities to realistic inoculation patterns reported from previous field studies, and compared the model output to the observed malaria prevalence in the field. There was overall consistency, supporting the potential of this agent-based methodology to represent transmission in realistic communities.

**Conclusions/Significance:**

Our approach represents a novel, convenient and versatile method to model *Plasmodium falciparum* infection.

## Introduction

Many attempts have been made to describe the complex in-host and population dynamics of malaria infection using mathematical models. Classical population-based models developed by Ross and MacDonald still provide the basis for many new approaches [1], [2], [3]. These models are based on SIR (Susceptible/Infected/Removed) methodology and sometimes aim at large-scale epidemiological predictions such as in a recent paper describing malaria dynamics in south-east Asia [4]. While any model may omit or simplify some aspects of reality, SIR are less adequate for infections like the one with *Plasmodium falciparum* [5]. Indeed, they allow only a minimalistic account of the complex immune processes within the human host. On a community level, SIR type models typically assume homogenous populations and host-vector interactions. Any kind of heterogeneity, such as multiple parasite strains and vector species, variable human characteristics (e.g. age, immunity and comorbidity), or type of intervention (e.g. drug treatment and bed net usage), will automatically increase the number of population strata and thus the number of variables and parameters defining the SIR system [6]. Mathematically, this leads to a substantial increase in the order and complexity of the system. However, only the simplest, low dimensional SIR models are amenable to algebraic manipulation and analysis.

Agent-based (AB) approaches can overcome some of these drawbacks. Using AB methodology, individual agents are represented by dynamic processes, describing in-host interactions of the malaria parasite with target cells and host immunity. A community of such agents can then be constructed and subjected to realistic transmission in the form of inoculation patterns. Unlike SIR systems, agent based (AB) communities are computationally constrained by their size since computing time and resources increase with population size. This limitation is, however, more than compensated by greater accuracy and versatility. For instance, an AB community can be made completely heterogeneous, allowing multiple parasite strains and/or species, human hosts with different age-dependent immunity and different interventions, at little or no additional computational cost. Several in-host models for malaria have been developed in previous studies [7], [8], [9], [10], [11], [12], [13], [14], [15]. They vary in scope and detail depending on their objective. Many focus on theoretical aspects of parasite interaction with the human immune system and the effect of antimalarial interventions. In some studies models were calibrated and validated using individual case clinical and parasitological data, such as for the first wave of asexual parasitemia [16], the full course of the infection based on informed trial and error [17], and the transition of asexual parasites to gametocytes [18], [19]. Several other studies have also applied the agent-based approach to a community level [20], [21], [22].

In the present study we developed a novel in-host agent model that accounts for the most salient features and biology of parasite-host interactions. This model and our calibration differ substantially from earlier work. In particular, we paid special attention to the parasite replication cycle (invasion and depletion of red blood cells (RBC), immune stimulation and parasite clearance. Mathematically, the model is implemented and run in discrete time steps based on the 48 h parasite replication cycle. Such discrete models behave in many aspects similar to models based on continuous differential equations, but they can be often implemented and simulated more efficiently, particularly for random processes. Our model combines deterministic and stochastic components of in-host dynamics, the latter resulting from random antigenic variation (AV) of *Plasmodium falciparum*. The model was coded in Wolfram Mathematica 7.

Our calibration procedure also differs from earlier related models [13], [17], [18], [19]. As in previous studies, we utilized individual host histories from malaria therapy (MT) records. However, we interpreted these MT histories in a different way. Rather than as an accurate benchmark for parameter fitting, we view each history as one of many possible random realizations of a stochastic AV process. Therefore, our calibration procedure combines deterministic and stochastic steps. Through fitting of the model to a large number of MT cases (n = 122), we created a pool of over 2000 parameter choices that serves as a basis for creating and simulating AB communities. We conducted several numeric experiments by subjecting these AB communities to realistic inoculation patterns as reported from malaria endemic regions. In particular, we studied the model predictions of malaria prevalence and compared them to the reported field observations.

## Methods

### Biological assumptions

Several important biological factors are included in our model of asexual parasitemia: (i) homeostatic production/loss of uninfected RBC; (ii) parasite replication (invasion of uninfected RBC and release of merozoites); (iii) stimulation of innate and adaptive immunity effectors by parasite density; (iv) parasite clearance by immune effectors.

**Figure 1:**
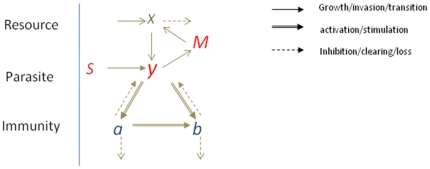
Schematic representation of the model. The population of uninfected red blood cells (*x*) provides the source for the infected population (*y*). Level I immune effector (*a*) is stimulated by y. Level II immune effector (*b*) is stimulated by *y* interacting with *a+b*. *M* represents the the number of merozoites, *S* represents an external source of inoculation.

A schematic view of the within-host processes is shown in Figure 1. We used the following notations: *x* – uninfected RBC population (per µL of whole blood), *y* – infected RBC population (per µL of whole blood), *a* – innate immune effector; *b* – adaptive immune effector.

The normal RBC level x_0_ (5×10^6^ µL^−1^) is maintained through stationary (homeostatic) production/loss terms. RBC are invaded by the newly released merozoite population (*M*). The probability of merozoite invasion depends on the available RBC pool per merozoite, *x/M*. The parasite burden stimulates an immune response consisting of innate and adaptive immune effectors *a* and *b*. We view these effectors as simplified proxies of the effector concentration (e.g., antibody titer) combined with the efficiency of the effector to clear infection. The immune effectors reduce parasite density by inhibiting parasite replication. Effectors are stimulated by parasite density, or through parasite-immune interactions above certain parasite density threshold levels.

The model was implemented using a discrete time-step based on parasite replication cycle rather than a process based on continuous differential equations. We utilize a novel approach to parasite antigenic variation (AV) and a novel calibration procedure.

*P. falciparum* has evolved several immune evasion strategies, most notably AV, whereby it can vary (on each replication cycle) an important class of surface proteins expressed on infected RBC. These proteins play a double role. On the one hand they serve as immunogenic targets with stimulation of antibody production and consequent parasite clearance. On the other hand they mediate adherence of infected RBC to endothelial cells in the microvasculature (sequestration) and thus promote parasite survival. AV of *P. falciparum* has been the subject of considerable research [23], [24], [25], [26], [27], [28], [29]. The process is controlled by the family of *var* genes. Each parasite genome contains 50–60 of these *var* genes [28]. During a replication cycle, each parasite expresses only one *var* gene but can switch expression in the next generation [30]. So a typical parasite population may include several antigenic variants present simultaneously. Therefore, every new infected RBC generation exhibits an altered antigenic profile compared to its predecessors, which has important implications for adaptive immunity. If some of the new variants are sufficiently distinct from their antecedents, the efficiency of previously developed adaptive immunity would be weakened [31].

There are different ways to account for AV in mathematical models. A direct approach to multi-variant parasite dynamics assigns different population variables to each variant. In addition, suitable ‘variability’ (exchange) patterns need to be set among multiple variants and their possible immune interaction with ‘specific effectors’ assigned to each type. These two processes are typically described by ‘mutability’ (switching) and ‘cross-reaction’ matrices. Such multi-dimensional approaches have been developed and utilized in previous studies (e.g. [19], [32]).

In the present study, we propose a simpler way to account for AV in a single infected RBC population. Assuming all variants are nearly identical in terms of growth and invasion, the only essential difference between them are their antigenic properties, i.e. susceptibility to previously developed adaptive immune effectors. As new infected RBC populations may differ antigenically from the earlier ones, the effective adaptive immune response may be reduced at each replication cycle. However, the magnitude of such a change should diminish with time as the parasite gradually depletes its repertoire of expressed *var* genes and the host develops antibodies against all antigenically distinct variants. We account for AV by *random falls* in adaptive effector *b* at each replication cycle. Randomness is essential for describing a typical ‘switching'/mutation’ process of any kind since, although they may not be truly random by nature, we typically lack detailed knowledge of their mechanisms and functions, and thus have to assume that they are random variables or parameters.

### Model setup

A definition of all variables and parameters used in this study is given in Table S1 in the supporting information. The basic system at time t (measured in reproductive cycles) is described by variables 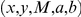: *x(t)* represents uninfected RBC (target cells) measured per µL of blood; *y(t)* represents infected RBC measured per µL of blood, 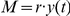 is the number of merozoites released; *a(t)* and *b(t)* are dimensionless variables representing innate and adaptive immune effectors. On each time step the transition from the current state 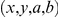 to the next state 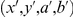 is given by the following equations.
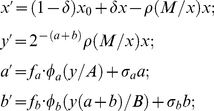
(1)The first two terms of the x-equation, 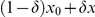, account for homeostatic production/loss of RBC that maintains its normal level *x_0_* = 5×10^6^/µL, with survival rate δ = 0.98/cycle (based on a 100 day life-span). The last term 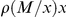 represents RBC loss due to merozoite invasion. Specifically, a density-dependent fraction 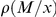 of *x* would be invaded and turned into the next generation of infected RBC (*y*′). Of those, only a fraction *2^−(a+b)^* would survive through to the end of the cycle, depending on the combined immune level *(a+b)*. Factor *r* in merozoite equation 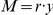 represents the *effective replication rate* of the parasite and is equal to the average merozoite progeny per infected RBC times the maximal probability of RBC invasion by a merozoite. The latter refers to optimal invasion conditions with large pool of available target cells (*x*), or a small relative merozoite population 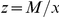. When fraction *z* grows large, merozoites start competing for available RBC, and the effective probability of invasion (or invaded fraction of *x*) decreases according to
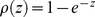
(2)Derivation of function 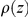 is based on two assumptions regarding the invasion process: (i) Poisson distribution of variable *z* about each (typical, average) RBC; (ii) exclusive competition where only one merozoite (among competing pool *z*) can establish successful invasion. This form of ‘invasion and resource depletion’ differs from the standard continuous formulation (e.g. [5], [8]), given by 2^nd^ order removal kinetics (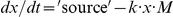).

### Immune regulation

Immune effectors *(a, b)* are dimensionless variables measured in terms of clearance of *y*, so that a ‘unit effector’ would halve the parasite population over the 2-day cycle (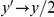). Both are stimulated by Hill functions 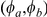 with suitable threshold transition levels and maximal stimulation efficiencies 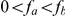. The innate stimulation 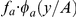 is triggered by the parasite level relative to threshold *A*, it has relatively short life-span (approximately 4 days) or survival rate 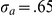, and lower efficiency *f_a_*. Adaptive effector (*b*) has longer memory (100 days or survival 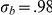) and higher clearing efficiency *f_b_*, but takes longer time to develop in naïve hosts. Furthermore, production of (*b*) in our model is triggered by the product of infected RBC density (*y*) and the combined effector pool *a+b* relative to threshold B. Product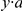 serves as a primary trigger for the development of adaptive responses, while 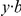 accounts for enhanced reactivation of *b* through adaptive immune memory. The efficiency factors (*f_a_*., *f_b_*.) represent the maximum stimulation rates of *a* and *b* respectively under ‘high’ parasitemia. The corresponding maximal clearance levels become
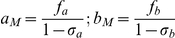
The latter imposes some constraints on model parameters to allow parasite clearance, namely
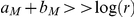
The numeric simulations of dynamic system (1) can result in arbitrarily low (unphysiological) parasite levels *(y)*. Therefore, we impose a lower cut-off on parasite density at *y_c_ = 10^−6^* µL^−1^ in our simulations, below which the parasite is considered cleared (*y = 0*).

### Antigenic variation (AV)

Equation system (1) represents the deterministic part of in-host processes. To account for AV, we let the adaptive immune effector (*b*) fall on each cycle by a random fraction 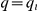 (0<q<1). Thus, the last equation of system (1) will take the form
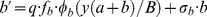
(3)The average severity of AV-induced reduction of *b* should diminish with each replication cycle, as the limited reservoir of antigenically distinct variants is depleted and the repertoire of host antibodies increases. In the present model we described this random process (*q* = Random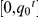) by an exponential function 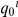 with base
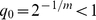
(4)dependent on the number of distinct variants and their cross-reactive properties, and represented by half-life parameter *m*. It should be noted that *m* does not represent the absolute number of variants. It is rather related to the number of antigenically distinct variant clusters. For example two var genes may encode for surface proteins which exhibit so much similarity, that antibodies developed specifically against one of them are effective to a certain degree against the other as well.

**Figure 2:**
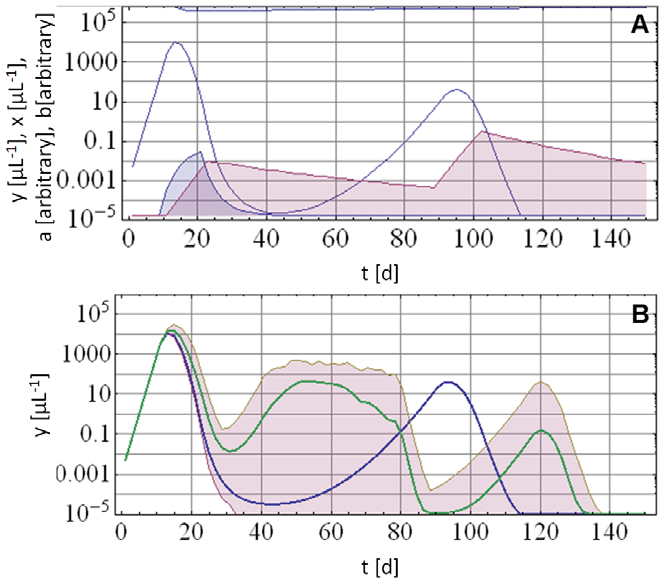
Deterministic pattern versus AV pattern. Panel A: Typical deterministic parasite density pattern (solid blue line) as predicted by the model. Also shown are innate immune effector *a* (blue filled curve) and adaptive immune effector *b* (purple filled curve). Panel B: Corresponding stochastically predicted mean parasite density (solid green line) and minimum/maximum envelope (purple fill) for the same deterministic solution (solid blue line) as shown in Panel A.

Figure 2 illustrates an example of a dynamic pattern resulting from the deterministic model (equation system (1)) and the corresponding ensemble made up of 50 random realizations of the stochastic AV process. We observe (Figure 2) that the deterministic component is dominant at early stages of infection (primary wave of parasitemia), while random AV variations become more pronounced later in the course of infection.

For numeric simulations and calibration of equation system (1), we fixed some model parameters, based on the available biological data and estimates and allowed others to vary. Tables 1 and 2 give a complete list of calibrated model parameters, and results of a sensitivity analysis. They include the effective replication factor *r*, innate and adaptive efficiencies (*f_a_*., *f_b_*.), immune stimulation thresholds *A* and *B* for *a* and *b* respectively and parameter *m* (related to the number of antigenically distinct clusters of variants).

**Table 1:** Possible ranges of uncertain parameters of the model, and medians and interquartile ranges which resulted from the fitting process.

Parameter	Name	Range	Median (25%/75% Quartiles)
Invasion probability	*p*	0.5–1	0.66 (0.57/0.82)
Replication	*r*	15–50	23.91 (18.66/30.94)
Innate efficiency	*f_a_*	5–20	7.59 (6.12/9.22)
Adaptive efficiency	*f_b_*	20–120	38.48 (28.73/49.23)
Innate threshold	*A*	30–80	66.57 (53.32/73.96)
Adaptive threshold	*B*	10–50	27.46 (18.37/37.39)
Antigenically distinct variant clusters	*m*	3–20	12 (6/14)

**Table 2:** Sensitivity analysis of in-host parameters and their contribution to dynamic patterns: strong ++; marginal +; no contribution −.

	Height 1^st^ peak	Day 1^st^ peak peak	height 2^nd^ peak	day 2^nd^ peak	clearing
*p*	++	+	−	−	−
*r*	++	+	−	−	−
*A*	−	−	−	−	+
*B*	−	−	+	++	−
*f_a_*	+	+	++	++	++
*f_b_*	−	−	++	++	++
*m*	−	−	++	++	++

Equation system (1) is appropriate for asexual stage dynamics in the absence of external infectious sources. External inoculations can be added to (1) by augmenting the merozoite variable
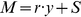
(5)with the source term *S* – representing the number of merozoites released from the liver per life cycle.

### Calibration procedure

The model was calibrated with data from MT studies for neurosyphilis, which provide a rare opportunity to examine host-parasite interaction over extended periods of time. The complete set of MT data used in the present study is given in Table S2 in the supporting information. These data have been analyzed in detail previously [33], [34], [35], [36] and used for calibration purposes or as ‘direct input’ for agent-based communities [13], [14], [22], [37].

The calibration procedure we propose involves two steps: a deterministic fit to the first wave of parasitemia, and a second stochastic step that attempts to accommodate irregular parasitemia patterns following the initial wave. Based on these steps we select ‘best choices’ of in-host parameters.

**Figure 3:**
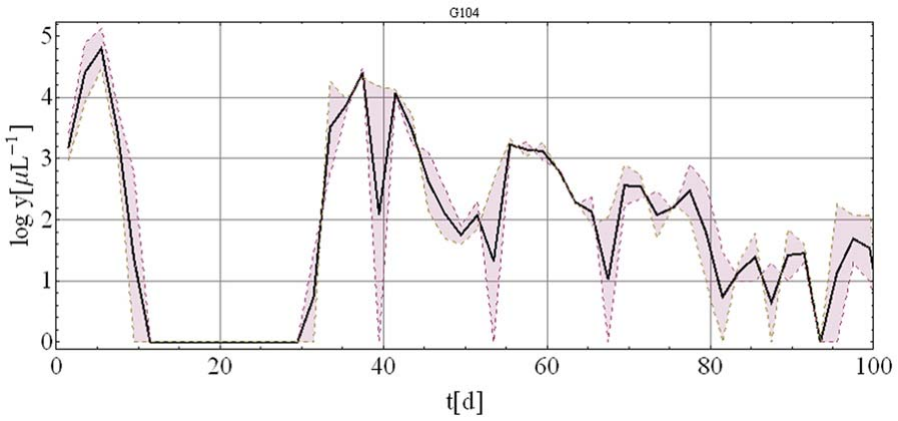
Typical MT-host with ‘odd-even’ envelope (purple shaded area) and its mean-curve (thick black line).

The total number of datasets available was for 334 MT patients. From these, we selected 122 for which patients were either untreated or treated very late in the infection. The MT patients exhibit highly irregular dynamic patterns of parasitemia for several reasons. Firstly, due to cytoadherence of late stage parasites there is an apparent oscillation with a 2-day recurring pattern. Since these oscillations have little relevance for long-term infection outcome, they were smoothed out by taking the maximum/minimum parasitemia envelopes on consecutive odd and even days, and computing their geometric mean curve, as illustrated by a representative patient history in Figure 3. On longer time scales there are recurrent irregular waves of parasitemia, often with diminishing amplitude. These recurrent waves are not a result of reinoculation (typical of a natural environment) as all MT hosts received a single initial inoculum in strictly controlled clinical experiments. Therefore these fluctuations in parasite density can be attributed to AV of *P. falciparum*, whereby new variants help to sustain infection over longer periods.

As MT hosts had no prior exposure to malaria, equation system (1) was run starting with the naïve initial state (*x_0_, y_0_, 0, 0*), i.e. normal RBC-level *x_0_*, initial inoculum *y_0_* = 0.001–0.01 µL^−1^, and no pre-existing immunity, 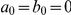. For calibration purposes the MT data set was divided into two groups: the first group consisted of cases that exhibited only a single wave of parasitemia, after which infection fell below the detection level (10 µL^−1^) and was presumed cleared. Such cases typically had a short duration of patent parasitemia (10–20 days). These cases were calibrated using the first (deterministic) step, as AV played no less important role in the initial wave of infection, confirmed by our numeric experiments.

The second group comprising long term irregular patterns with multiple waves of parasitemia was subjected to two calibration steps. In the first step, we collected 50 of the best-fit deterministic parameters (*r, A, B, f_a_, f_b_*) (Table 1) from each suitable MT dataset and a random ensemble of 25000 parameter choices. The ‘best-fit’ was confined to the first wave of parasitemia, and we used the standard square-mean error between the observed and simulated histories
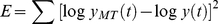
(6)As the MT data does not inform on inoculation dates, we estimated the time lapse *T* between inoculum and the first day with observed parasite density and adjusted it in the fitting process along with other parameters of Table 1. In the second calibration step (for multiple-wave patterns) the best-fit parameter choices of step 1 were further adjusted to account for an extended history and random AV effects. The adjustment involved only immune efficiencies (*f_a_, f_b_*), as those are primarily responsible for the long term pattern of parasitemia and have only a minor effect on the primary wave of parasitemia (Table 2).

We consider MT histories to be random realizations of a stochastic AV process rather than unique ‘individual patterns’. Therefore if the same host would be subjected to another inoculation he/she may exhibit a different infection pattern. In general, it is a challenging task to calibrate parameters of a stochastic process from its single realization and obtain statistically reliable results. The standard techniques would typically require a sufficiently ‘long history’ and ‘simple stochasticity’ (e.g. linear stationary process with ‘additive noise’). In the present model, we are dealing with a highly nonlinear system (1) and non-stationary, multiplicative noise, since the AV input is a decaying random sequence 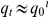. Our approach is to create, for each adjusted choice 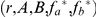 with 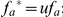 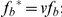 1<*u*<2 and 1<*v*<4, a random AV-ensemble of 50 realizations, then try to fit a given MT history within the ‘min/max ensemble envelop’ by minimizing its distance from the ensemble mean curve (all y-values are log-transformed). The best-choice values of factors (u*,v*) and the adjusted 5-tuples 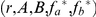 are then selected to represented a given host.

The computational codes were implemented and run in Wolfram Mathematica 7. The code can be downloaded from: http://www.cwru.edu/artsci/math/gurarie/Malaria/In host calibration.nb (code) and http://www.cwru.edu/artsci/math/gurarie/Malaria/Sorted%20mean%20hist%20210 (filtered MT data in Mathematica format).

The Mathematica codes and procedures developed for the model are very efficient and require only modest computational resources. The efficiency is important for our calibration procedures (computing large ensembles of hosts and histories) and applications to agent based communities (AB communities).

### Agent Based Communities

AB communities were assembled from the best parameter sets resulting from the calibration process (50 best parameter sets for each calibrated MT data set). The AB communities used in the present study typically consisted of 1000 agents. The communities (agents) were subjected to external inoculation (via the *S*-term in Equation (6)), based on entomological inoculation rates (EIR) as observed in field studies. External inoculation will produce different recurrent patterns that combine in-host regulation with external forces. In the present study, the inoculation patterns were generated as series of binaries (0 and 1) with 1 being an inoculum. The probability that an inoculation occurs on a specific time step is dependent on the EIR, which represents mean number of inoculations per agent in a specific time interval. Each agent was subjected to an individual inoculation pattern based on the same average EIR.

Model predictions were compared with the data from three selected reports from Africa that correlated observed temporal malaria prevalence patterns with observed EIRs. The reported EIR patterns were used as input for the AB community model and the model output compared to the data reported from these studies. The first study was by Beier et al. from 1999 [38]. It provided an overall correlation of EIR with malaria prevalence in Africa in the form of a review combining data from different study sites. To compare model predictions with these data, the AB community was subjected to stationary biting sequences (determined by EIR) which corresponded to those reported in the study by Beier. The two other studies were by Vercruysse et al. 1983 [39] and Gazin et al. 1988 [40]. Both correlated EIR and malaria prevalence in seasonal transmission environments. The former study of these two was conducted in an urban area of the Senegal, the city of Pikine in 1979–1981. EIRs were reported in monthly intervals from December 1979 to December 1980. Parasite prevalence rates were reported at 7 time points, the first being in November 1979 the last in January 1980, so that 2 of the 7 data points fall outside the range in which EIR was measured. Gazin et al. conducted their study in the village of Kongodjan in Burkina Faso. Transmission there was also seasonal but the prevalence was much higher than in the study by Vercruysse et al. EIRs were reported in monthly intervals from January 1983 to January 1985. Parasite prevalence was reported in 10 intervals from December 1982 to March 1985. For both comparisons, it was assumed that the EIR observed at one time point changed linearly to that at the next time point. In both studies, the reported EIR had the unit infective bites/day but, in the model, this was converted to infectious bites per asexual life cycle (48 h) by multiplication by 2. The reported EIR patterns were then used as an input for the AB community model. The malaria prevalence predicted by the model was plotted in comparison to the reported prevalence.

## Results

### Deterministic and stochastic patterns produced by the model

**Figure 4:**
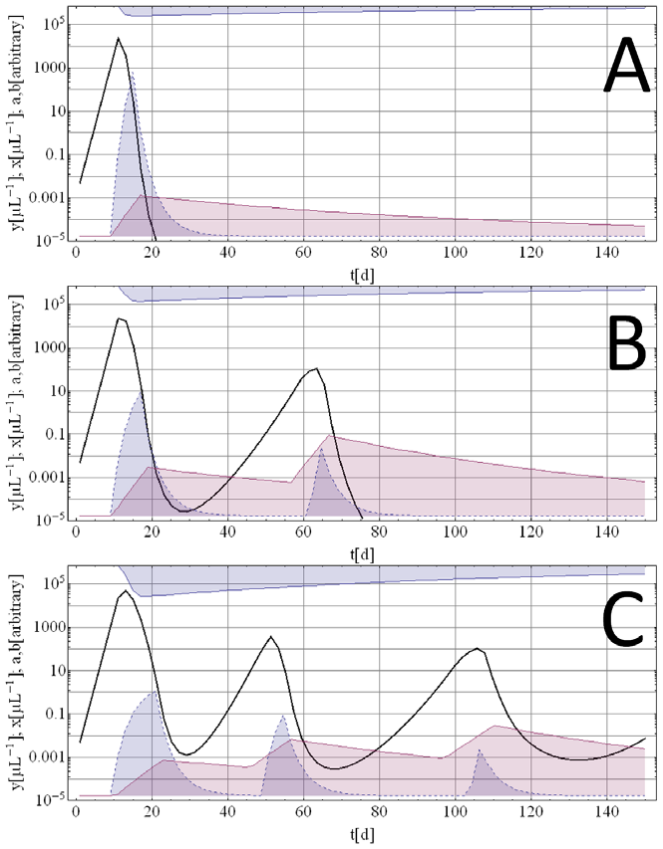
Typical deterministic histories starting from an immunologically naïve state with an initial inoculum. Blue solid lines are parasitemia, the blue filled curve is immune effector *a*, the purple filled curve immune effector *b*, and the blue filled curve at the top are depleted resource cells. Deterministic histories can have single (Panel A), double (Panel B) and multiple (Panel C) wave patterns. However multiple waves patterns very rarely terminate and look very different from the MT data.

Depending on the parameters, the model can exhibit diverse dynamic patterns of infection. The main patterns are shown in Figure 4 in which the simulations depicted use one initial inoculum into a naïve host. The deterministic oscillations result primarily from the gradual decline of innate and adaptive immune effectors *a* and *b* while the parasite density remains low but above the cut-off threshold. As *a* and *b* weaken further, the restraints on parasite growth diminish. Typically, the second and subsequent waves will be much lower in parasite density and further expansion of *b* during recrudescent phases may eventually clear the infection. While the deterministic model can produce multiple oscillations for certain parameter values, such deterministic waves look very different from the observed MT cases. This observation confirmed our hypothesis that simple deterministic models cannot account for the complexities of MT cases, and that the proper calibration procedure would require an additional stochastic component. We used AV as stochastic component as proposed in the Methods section.

Figure 2 demonstrates the principal effect of applying the stochastic AV process to the deterministic model. Figure 2A shows an example of a deterministic curve resulting from a specific set of parameters. An example for an adjusted AV ensemble of 50 random realizations with its mean parasite density pattern and minimum/maximum envelope is shown in Figure 2B. The AV modified patterns resemble the MT data more closely. After the initial growth phase, which is nearly identical for deterministic and AV modified model predictions, AV retards the accumulation of protective immunity. Hence there are delayed and higher parasitemia peaks for the AV modified model predictions. For instance, in Figure 2 where *y_max_* = 2×10^3^ at day 13 for the deterministic pattern (Panel A), *y_max_* = 2*10^3^ to 3*10^3^ at days or 13 to 15 for AV modified patterns (Panel B).

### Calibration of the model using MT Data

**Figure 5:**
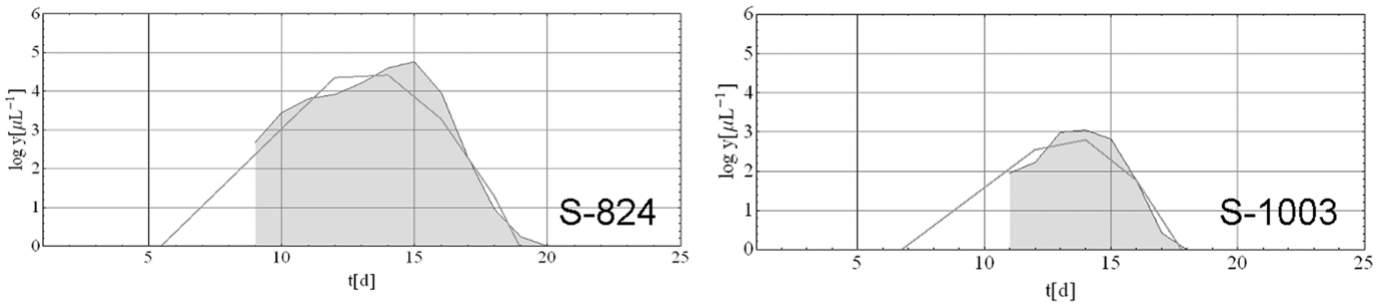
Two typical single wave datasets. The solid gray lines are parasitemia. The curve with light gray fill is the model prediction.

Twenty-five datasets used for calibration in this study exhibited single wave patterns of parasitemia. Figure 5 shows two typical fitted simulated patterns along with the original MT data from patients who had a single wave of parasitemia. A large number of these fits can be found in the supporting information (Figures S1, S2, S3, S4, S5, S6, S7, S8, S9, S10) In terms of duration, maximum parasite density and day of maximum parasite density, the deterministic calibration resulted in good curve fits for most single wave MT cases.

For irregular patterns of longer duration, the qualities of the fits varied. Although AV, as modeled here, can account for a large number of irregular cases, some MT patterns are entirely different and fall outside the current setup. A typical stochastic pattern produced by our model exhibits an initial wave of parasitemia to a peak, with a subsequent decline until clearance.

**Figure 6:**
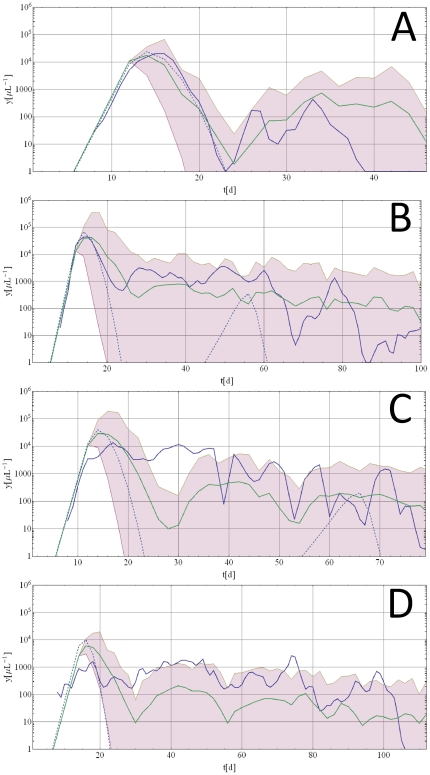
Long term, multiple wave datasets calibrated with the present model. Panels A and B depict cases where the calibration resulted in a reasonable fit. The datasets are suitable because they exhibit an initial wave of parasitemia which contains the global maximum of the entire history. Panels C and D depict cases which are less suitable because they are missing a pronounced first wave of parasitemia. The blue solid lines are the original MT data, the dashed blue lines are the best fits to the first wave of parasitemia (1st calibration step), the purple shaded areas are the AV envelopes (2nd calibration step) with its mean curve (solid green line).

Figure 6 shows stochastic data fits resulting from the two-step calibration method for hosts with irregular parasitemia. The fits in Panels A and B are acceptable on two counts. First, the MT-data are contained entirely within the envelope of the AV-ensemble. The ensemble envelope represents the range of possible simulated stochastic patterns and can therefore be viewed as a measure of the ensemble variation about its mean. Second, the MT data pattern falls within a reasonable range of the mean pattern of the simulated ensemble. This is the case for most of the 97 irregular patterned datasets calibrated with the two step method. Panels C and D show two data fits of irregular patterns that exhibit greater departure from the model behavior. The dataset shown in Panel C (S-1249) starts with a plateau of parasitemia, while in most model simulations we see a typical ‘initial wave’ of shorter duration. The dataset shown in panel D (S-713) exhibits an irregular (fluctuating) initial growth stage. While these cases depart from the ‘model pattern’ at the early stage, their AV-envelops representing stochastic patterns based on the 50 best parameter sets still cover the remaining (long-term) trend reasonably well. The majority of datasets (71/97) exhibited basic characteristics in concordance with model output. Graphic representations of many of these fits can be found in the supporting information (Figures S11, S12, S13, S14, S15, S16).

**Figure 7:**
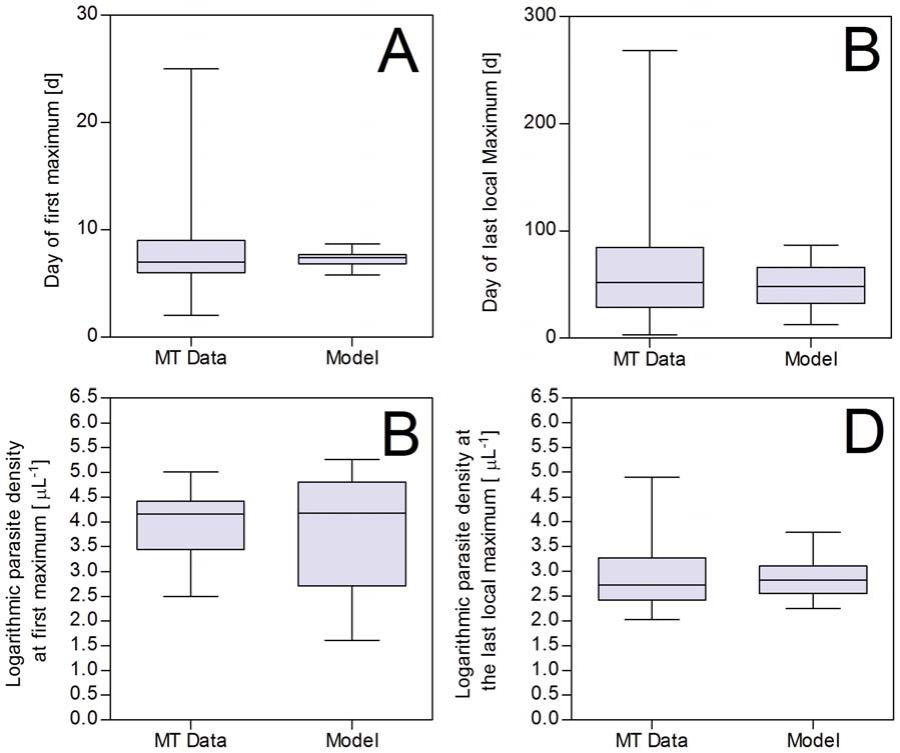
Comparison of characteristic statistics between MT data and model prediction. Panel A: Day of the first maximum; Panel B: Day of the last maximum; Panel C: Parasite density at first maximum; Panel D: Parasite density at last maximum; None of these characteristic features where significantly different between model and MT data.

For each dataset, parameter ensembles were collected that resulted in the best fits of parasitemia patterns of MT patients. We compared characteristic statistics (specifically the days and parasite densities of the first and the last maximum) of the MT data set with the same predicted characteristics from of a community generated using the best parameters collected for each MT dataset in the calibration process. The results of this comparison are presented in Figure 7. A comparison to characteristic features of infection patterns used in a previous study is included in the supporting information (Table S3) [13].

### Agent based communities

As a first test of validity of our calibration procedure, we created two AB communities. The first AB community was built from our collection of fitted parameters the second from random parameter choices within the calibration ranges (Table 1). The predicted parasite prevalence using calibrated parameter sets differed considerably from the predictions using random parameter sets over a wider range of EIR. Figure S17 in the supporting information illustrates the observed differences. Thus we conclude that MT calibration does provide a meaningful selection of model parameters, in a statistical sense.

**Figure 8:**
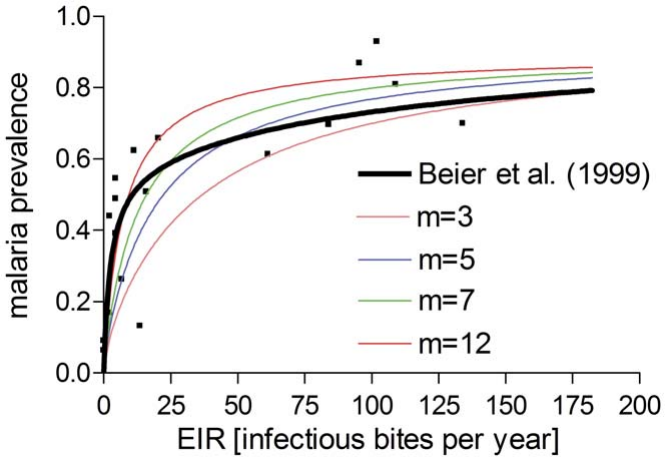
AB communities subjected to stationary EIR and comparison to field data. The data points (black) are taken from a review conducted by Beier et al. (1999) [33]. The black line is the curve fit also given in that reference. The colored lines are model predictions based on different numbers of antigenically distinct variant clusters (*m*).

**Figure 9:**
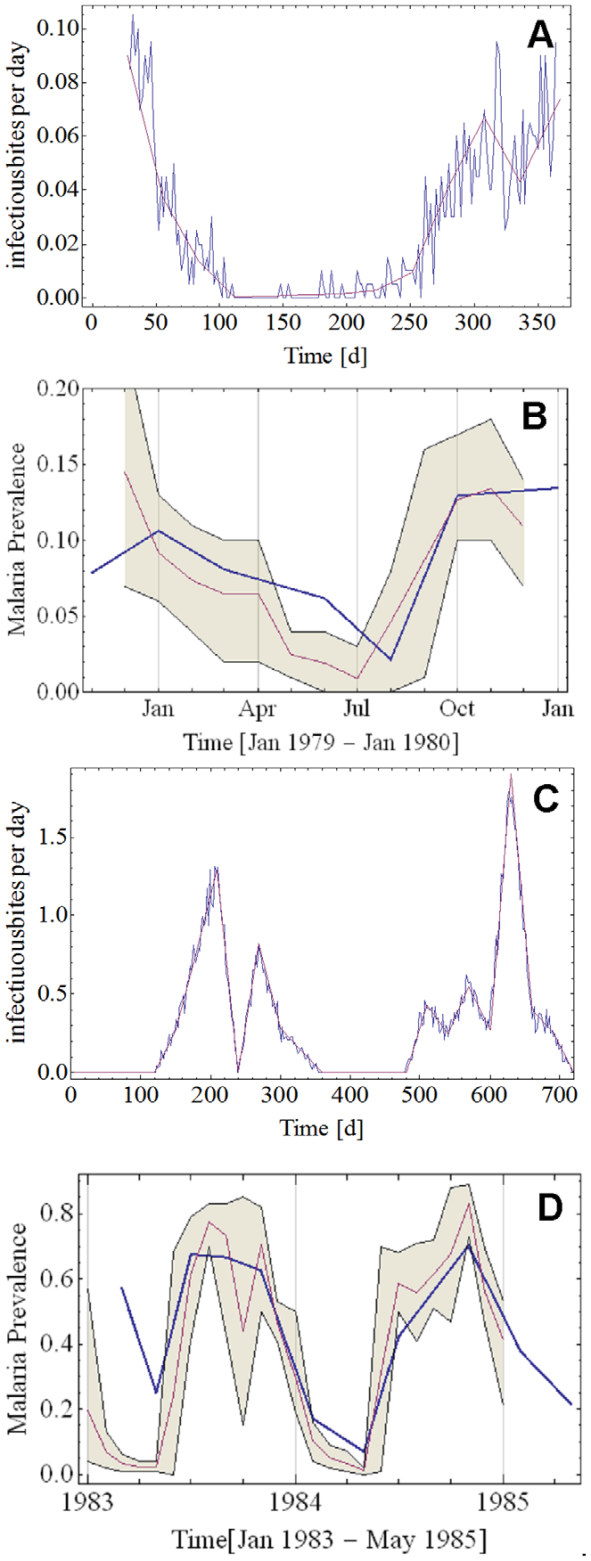
Comparison of model prediction to field observations from areas of seasonal malaria transmission. Panel A: EIR as reported by Vercruysse [34] (solid purple line), and reproduced as input for the model (solid blue line). Panel B: Malaria prevalence as reported by Vercruysse (solid blue line) and model prediction as monthly average (solid purple line) and envelope of monthly minima and maxima (olive fill) using as input the EIR pattern from Panel A. Panel C: EIR as reported by Gazin [35] (solid purple line), and reproduced as input for the model (solid blue line). Panel D: Malaria prevalence as reported by Gazin (solid blue line) and model prediction as monthly average (solid purple line) and envelope of monthly minima and maxima (olive fill) using as input the EIR pattern from Panel C.

The comparison with the data from Beier et al 1999 [38] is shown in Figure 8. There is a good agreement between reported and predicted values, however our range of EIR was limited to 182.5 based on 2-day cycle) compared with >700 per year as recorded in the publication. The comparison with the studies of Vercruysse et al. 1983 [39] and Gazin et al. 1988 [40] are shown in Figure 9. Figure 9 A and B show the observed and simulated EIR pattern, and the observed and predicted malaria prevalence for the comparison with the data from Vercruysse et al. 1983 [39]. Figure 9 C and D show the same data for the comparison with the study of Gazin et al. 1988 [40]. There is good agreement between the observed and predicted parasite prevalence dynamics over a wide range of time points.

## Discussion

AB modeling of malaria requires the construction of a reasonable in-host model as a foundation. Depending on its intended use, the model is a compromise between reasonable simplicity and the complex mechanisms it seeks to describe on the in-host epidemiological scales. In this report, we present an agent based model that can be used to reproduce MT data with reasonable accuracy and is computationally efficient so that communities of 1000 or more agents can be run on a desktop computer without a long processing time.

Several types of within-host malaria models have previously been developed [3], [12]. Some use continuous differential equations, with very limited account of immunity and make no attempt to accommodate AV or fit unknown parameters [7], [8], [9], [10], [11]. Other studies have developed models which use a discrete-time step approach for single or multi-variant parasite densities [13], [14], [16], [17], [18]. Multi variant modeling approaches typically require more computational power and involve larger sets of uncertain parameters for model calibration. It might be difficult to relate their setup and output to epidemiological data and considerations. Besides, community level effects may become insensitive to the detailed structure of multi-variant systems.

Some previous models were fitted to MT data by either formally fitting specific characteristic features (e.g. the first-wave of parasitemia, duration of infection, maximum parasitemia, various slopes, number of local maxima, *etc.*) or using informed trial and error methodology [13], [16], [17].

To our knowledge, no previous study has developed a formalism to fit the entire length of MT histories and utilized the resulting fitted parameters to generate AB communities for simulations and data analysis. We proposed a novel calibration method in this study that differs considerably from earlier approaches. We assumed that all parasitemia patterns beyond the first wave are the result of a stochastic AV process with multiple uncertain contributing factors. Therefore, each of these patterns is a single realization of the process and subjecting the same host to another inoculation with the same parasite and the same number of sporozoites may result in a different pattern of parasite density. However we also assumed that these stochastic patterns fall within certain boundaries. Ideally, a calibration procedure for stochastic patterns should use stable output characteristics and statistics, and reconstruct the unknown model parameters based on them. In case of the MT data, these output characteristics could be the duration of the infection, the day of maximum parasite density, the maximum parasite density and so on. These outputs are, however, themselves random quantities. Thus, to give a statistically reliable prediction, such a calibration procedure would require more than a single individual realization of the stochastic process. The MT data do not provide this information since each patient was inoculated only once. The standard methods of parameter estimation for stochastic processes are inappropriate in our context, as the equations (1)–(4) are nonlinear, and randomness (AV) enters in a complicated nonlinear fashion. Therefore, we proposed that MT patterns are random realizations of the stochastic process and tried to ‘optimally’ fit them within a suitable ensemble envelope.

The majority of the MT datasets exhibit a dominant first wave of parasite density followed by recurrent, diminishing waves. Our calibration procedure resulted in reasonable fits for all MT datasets exhibiting these basic features. We validated our scheme by comparing AB communities based on our calibrated parameter sets with purely random parameter choices, and found substantial differences in their predicted outputs (Figure S17 in the supporting information). Furthermore characteristic features in the MT data were reproduced reasonably well by the model. We therefore concluded, that the calibration to a certain extent allows a meaningful selection of in-host parameters. We then subjected the calibrated AB community to random inoculations at prescribed rates (EIR) based on several field studies and found reasonably good agreement.

There are several limitations in our approach and results. On the in-host side, it may be desirable to account for variant diversity and more detailed structure of immunity. In the current setup all variants were considered to have identical growth and clearance characteristics. Furthermore, the immune regulation takes an abstract form represented by only two effector variables (*a* and *b*). A further extension of the in-host model would include more detailed structure of the immune system with different B- and T-cell populations and relevant processes (activation, proliferation, effector function, parasite clearing and memory maintenance), along with antibodies, cytokines and fever.

Another limitation of the present model is the 2-day replication cycle, which limits the dynamics process and specific features of different (young/mature) parasite stages. Furthermore we modeled only a single parasite strain and a useful extension of the present model would include multiple strains with different fitness and drug susceptibility.

The calibration procedure was limited mostly by the availability of data. Ideally, unperturbed (baseline) parasite density patterns from populations exposed to different endemicity levels and with different ages should be used to calibrate the model. However such data are not available. It remains to be determined whether MT-based calibration results are applicable to field data and how it should be modified to account for the important (e.g. individual or age-dependent) differences in malaria infection.

A major limitation of the AB community part of the present model is that there is no transmission. Therefore, the AB community is assumed to be a very small part of a larger transmission environment and changes in malaria prevalence within the AB community have no effect on this transmission environment. Future model developments and applications will include gametocyte production as a function of the asexual parasite density and calibrate gametocyte density using MT data or field data [41].

When comparing model predictions to observed field data it should be noted that the calibration proposed in the present study was conducted using data from adults who had never been exposed to malaria before. In reality, this is almost never the case. In the studies by Vercruysse and Gazin, only children were enrolled and we have to assume that nearly all of these children had been exposed to malaria before these studies commenced. Furthermore there was considerable usage of bed nets, chemoprophylaxis and insecticide spraying over the course of these studies. These are factors that are not taken into account by the present model. Extended versions of the model should address these issues. Nevertheless, it is evident that the model predictions and the observed prevalences are very similar over a wide range of compared data. We therefore concluded that this model can make some useful predictions and serve as a basis for future development.

## Supporting Information

Figure S1 **Graphic representations of the six best fits to the first wave of parasitemia for datasets 35, 37, 38, 39, 40, 41, 42 and 43 (A–H).** X axes are days, y axes are decadic logarithms of parasite density. The numbers above the graphs are the errors calculated using equation (6).
(TIF)

Figure S2 **Graphic representations of the six best fits to the first wave of parasitemia for datasets 44, 45, 46, 48, 50, 51, 52 and 54 (A–H).** X axes are days, y axes are decadic logarithms of parasite density. The numbers above the graphs are the errors calculated using equation (6).
(TIF)

Figure S3 **Graphic representations of the six best fits to the first wave of parasitemia for datasets 55, 56, 57, 58, 59, 60, 61 and 62 (A–H).** X axes are days, y axes are decadic logarithms of parasite density. The numbers above the graphs are the errors calculated using equation (6).
(TIF)

Figure S4 **Graphic representations of the six best fits to the first wave of parasitemia for datasets 63, 64, 67, 69, 70, 71, 73 and 74 (A–H).** X axes are days, y axes are decadic logarithms of parasite density. The numbers above the graphs are the errors calculated using equation (6).
(TIF)

Figure S5 **Graphic representations of the six best fits to the first wave of parasitemia for datasets 76, 77, 78, 79, 80, 81, 82 and 83 (A–H).** X axes are days, y axes are decadic logarithms of parasite density. The numbers above the graphs are the errors calculated using equation (6).
(TIF)

Figure S6 **Graphic representations of the six best fits to the first wave of parasitemia for datasets 84, 85, 86, 87, 88, 89, 90 and 91 (A–H).** X axes are days, y axes are decadic logarithms of parasite density. The numbers above the graphs are the errors calculated using equation (6).
(TIF)

Figure S7 **Graphic representations of the six best fits to the first wave of parasitemia for datasets 92, 93, 94, 95, 96, 97, 98 and 99 (A–H).** X axes are days, y axes are decadic logarithms of parasite density. The numbers above the graphs are the errors calculated using equation (6).
(TIF)

Figure S8 **Graphic representations of the six best fits to the first wave of parasitemia for datasets 100, 101, 102, 103, 105, 106, 107 and 109 (A–H).** X axes are days, y axes are decadic logarithms of parasite density.
(TIF)

Figure S9 **Graphic representations of the six best fits to the first wave of parasitemia for datasets 110, 111, 114, 115, 116, 117, 118 and 119 (A–H).** X axes are days, y axes are decadic logarithms of parasite density. The numbers above the graphs are the errors calculated using equation (6).
(TIF)

Figure S10 **Graphic representations of the six best fits to the first wave of parasitemia for datasets 120 and 121 (A–B).** X axes are days, y axes are decadic logarithms of parasite density. The numbers above the graphs are the errors calculated using equation (6).
(TIF)

Figure S11 **Best ensemble fits to the entire course of infection for data sets 37, 39, 40, 41, 44, 45, 46, 48, 50, 51, 52 and 54 (A–L).** Blue lines are the MT data, green lines are the ensemble means and shaded purple areas are ensemble envelopes. X axes are days, y axes are decadic logarithms of parasite density.
(JPG)

Figure S12 **Best ensemble fits to the entire course of infection for data sets 55, 56, 57, 58, 59, 60, 62, 63, 64, 67, 69 and 70 (A–L).** Blue lines are the MT data, green lines are the ensemble means and shaded purple areas are ensemble envelopes. X axes are days, y axes are decadic logarithms of parasite density.
(TIF)

Figure S13 **Best ensemble fits to the entire course of infection for data sets 71, 73, 74, 76, 77, 78, 79, 80, 81, 82, 83 and 84 (A–L).** Blue lines are the MT data, green lines are the ensemble means and shaded purple areas are ensemble envelopes. X axes are days, y axes are decadic logarithms of parasite density.
(TIF)

Figure S14 **Best ensemble fits to the entire course of infection for data sets 85, 86, 87, 88, 89, 90, 91, 92, 93, 94, 95 and 96 (A–L).** Blue lines are the MT data, green lines are the ensemble means and shaded purple areas are ensemble envelopes. X axes are days, y axes are decadic logarithms of parasite density.
(TIF)

Figure S15 **Best ensemble fits to the entire course of infection for data sets 97, 98, 99, 100, 101, 102, 103, 105, 106, 107, 109 and 110 (A–L).** Blue lines are the MT data, green lines are the ensemble means and shaded purple areas are ensemble envelopes. X axes are days, y axes are decadic logarithms of parasite density.
(TIF)

Figure S16 **Best ensemble fits to the entire course of infection for data sets 111, 114, 115, 116, 117, 118, 119, 120 and 121 (A–I).** Blue lines are the MT data, green lines are the ensemble means and shaded purple areas are ensemble envelopes. X axes are days, y axes are decadic logarithms of parasite density.
(TIF)

Figure S17 **6 panels comparing random versus best parameter based community predictions of the model.** The panels on the left hand side are community runs using random parameters. The panels on the right hand side are community runs using parameters from the model calibration. Community size is n = 2000. Panels A and B compare the community prevalences at an EIR of 1 per parasite reproductive cycle (182.5 per annum), Panels C and D compare the community prevalences at an EIR of 0.1 per cycle (18.3 per annum), and panels E and F compare community prevalences at an EIR of 0.01 per cycle (1.83 per annum). The dotted black lines denote fraction of uninfected RBC, the dashed black denotes iRBC, and the solid gray denotes infected but below limit of detection by light microscopy (10 parasites per microliter).
(TIF)

Table S1 **Description of all variables, parameters and indices used in the model.**
(DOC)

Table S2 **Overview over the MT data used in the present study and allocation of numbers to each set of MT data.** The columns labeled ‘#’ are the numbers assigned to each dataset over the course of this study to facilitate data processing.
(DOC)

Table S3 **Comparative statistics between the output of our model, the MT data we used for calibration and data presented by Gatton et al 2006** [**13**] **.**
(DOC)
