# Supplementary figures and images for: Mathematical Modeling of Malaria Infection with Innate and Adaptive Immunity in Individuals and Agent-Based Communities
Source: PLoS One. 2012 Mar 28;7(3):e34040. doi: 10.1371/journal.pone.0034040 (PMC3314696; doi:10.1371/journal.pone.0034040)

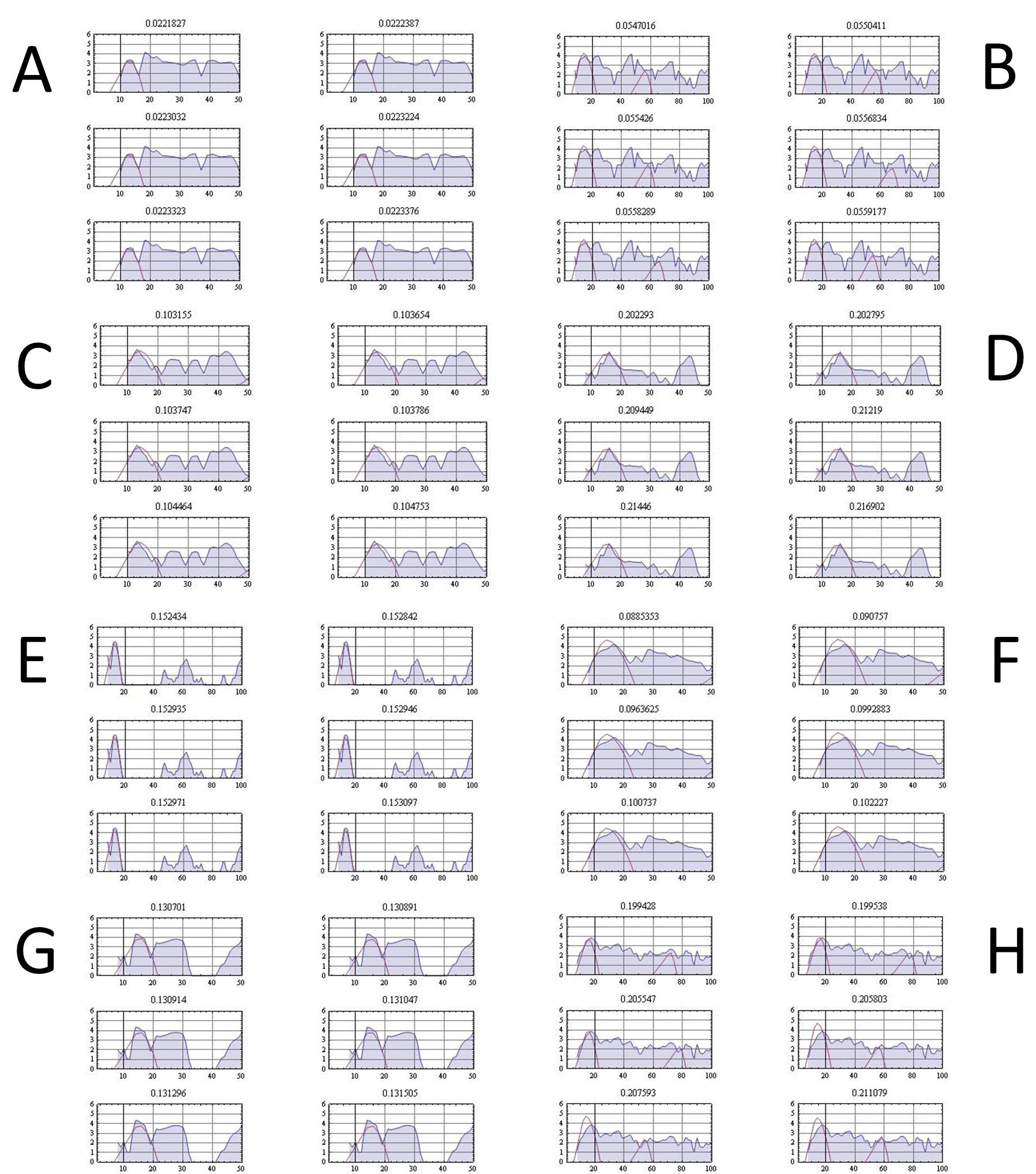

Supplement: Figure S1 — Graphic representations of the six best fits to the first wave of parasitemia for datasets 35, 37, 38, 39, 40, 41, 42 and 43 (A–H). X axes are days, y axes are decadic logarithms of parasite density. The numbers above the graphs are the errors calculated using equation (6). (TIF) [file pone.0034040.s001.tif]

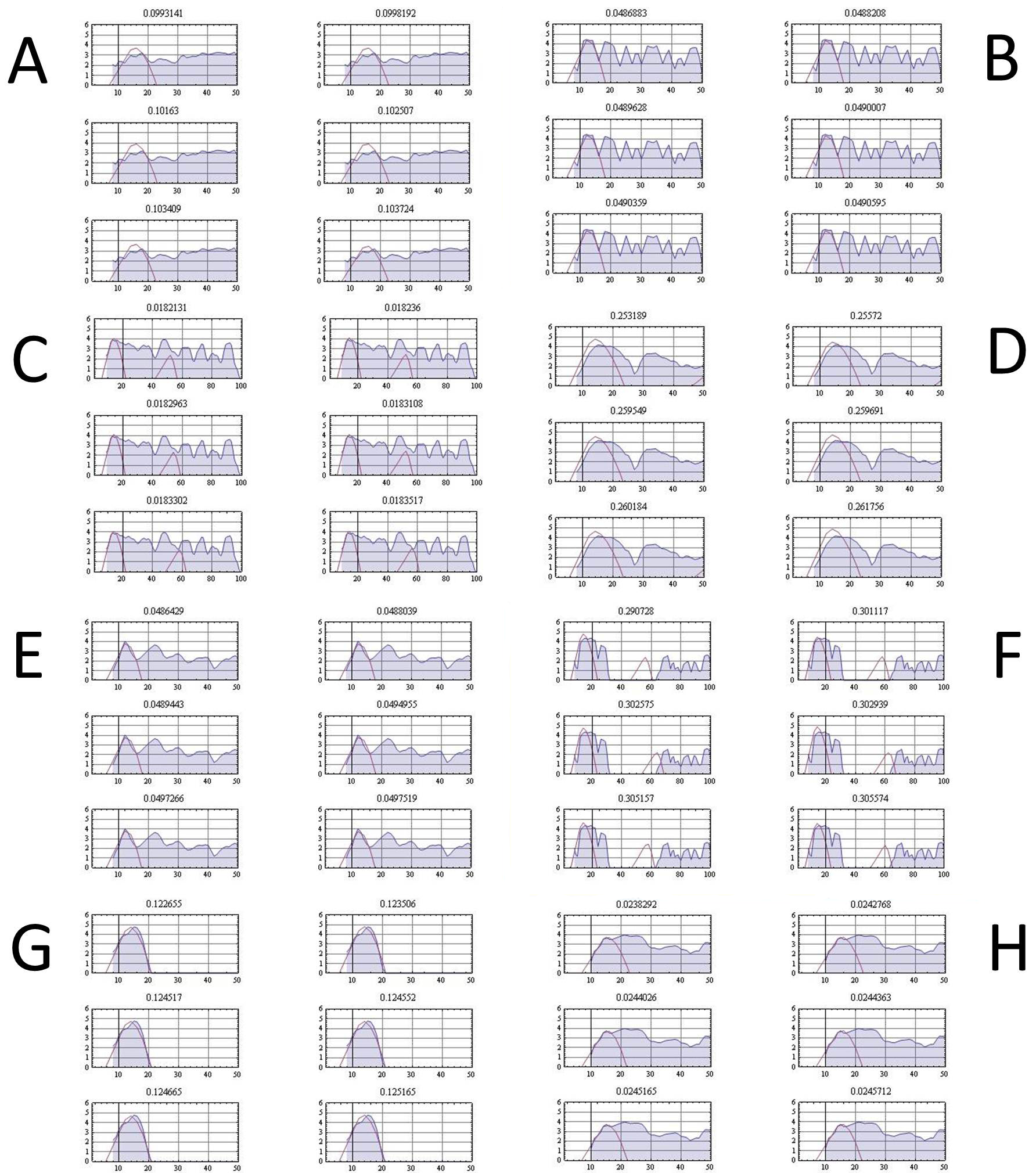

Supplement: Figure S2 — Graphic representations of the six best fits to the first wave of parasitemia for datasets 44, 45, 46, 48, 50, 51, 52 and 54 (A–H). X axes are days, y axes are decadic logarithms of parasite density. The numbers above the graphs are the errors calculated using equation (6). (TIF) [file pone.0034040.s002.tif]

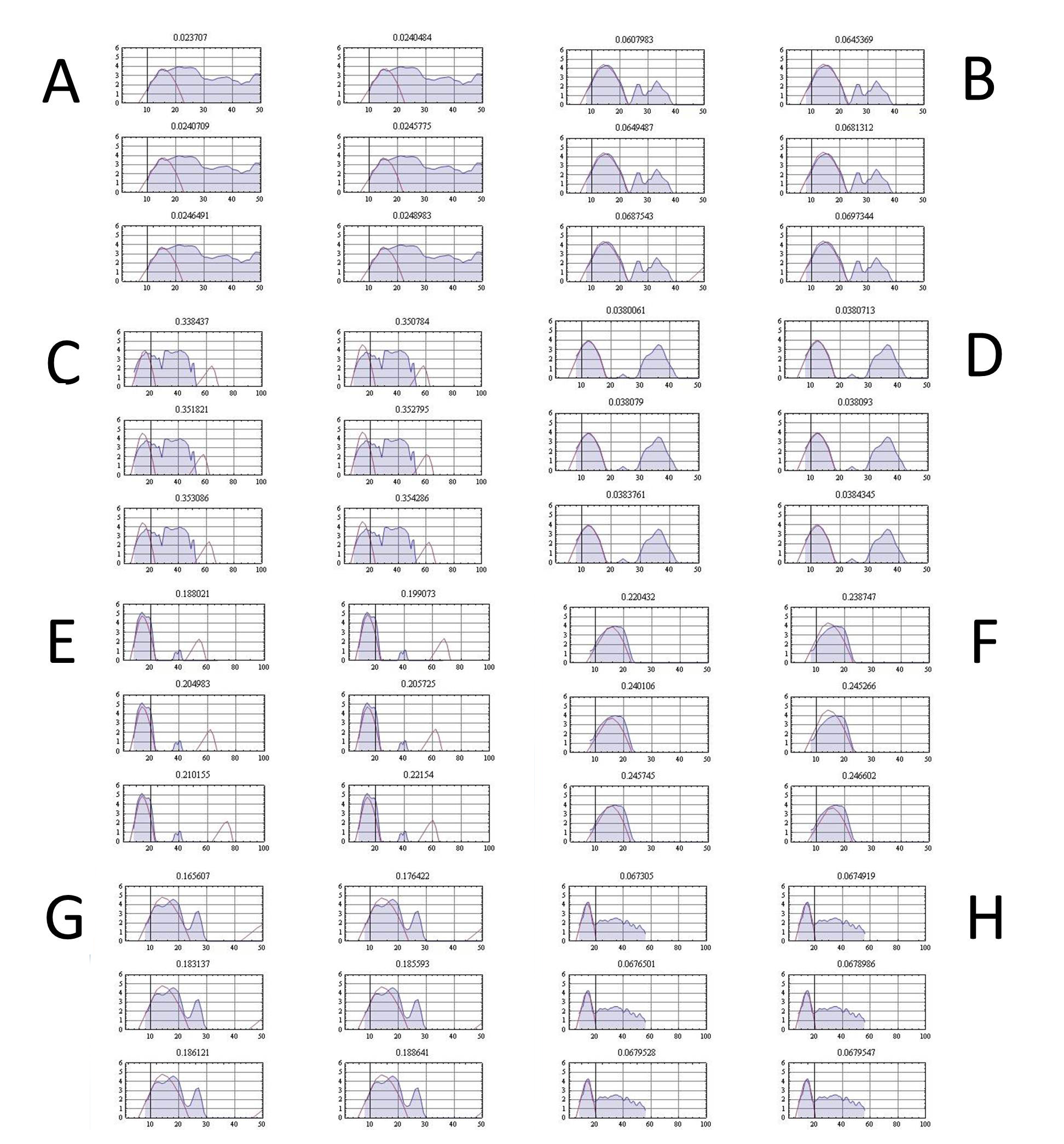

Supplement: Figure S3 — Graphic representations of the six best fits to the first wave of parasitemia for datasets 55, 56, 57, 58, 59, 60, 61 and 62 (A–H). X axes are days, y axes are decadic logarithms of parasite density. The numbers above the graphs are the errors calculated using equation (6). (TIF) [file pone.0034040.s003.tif]

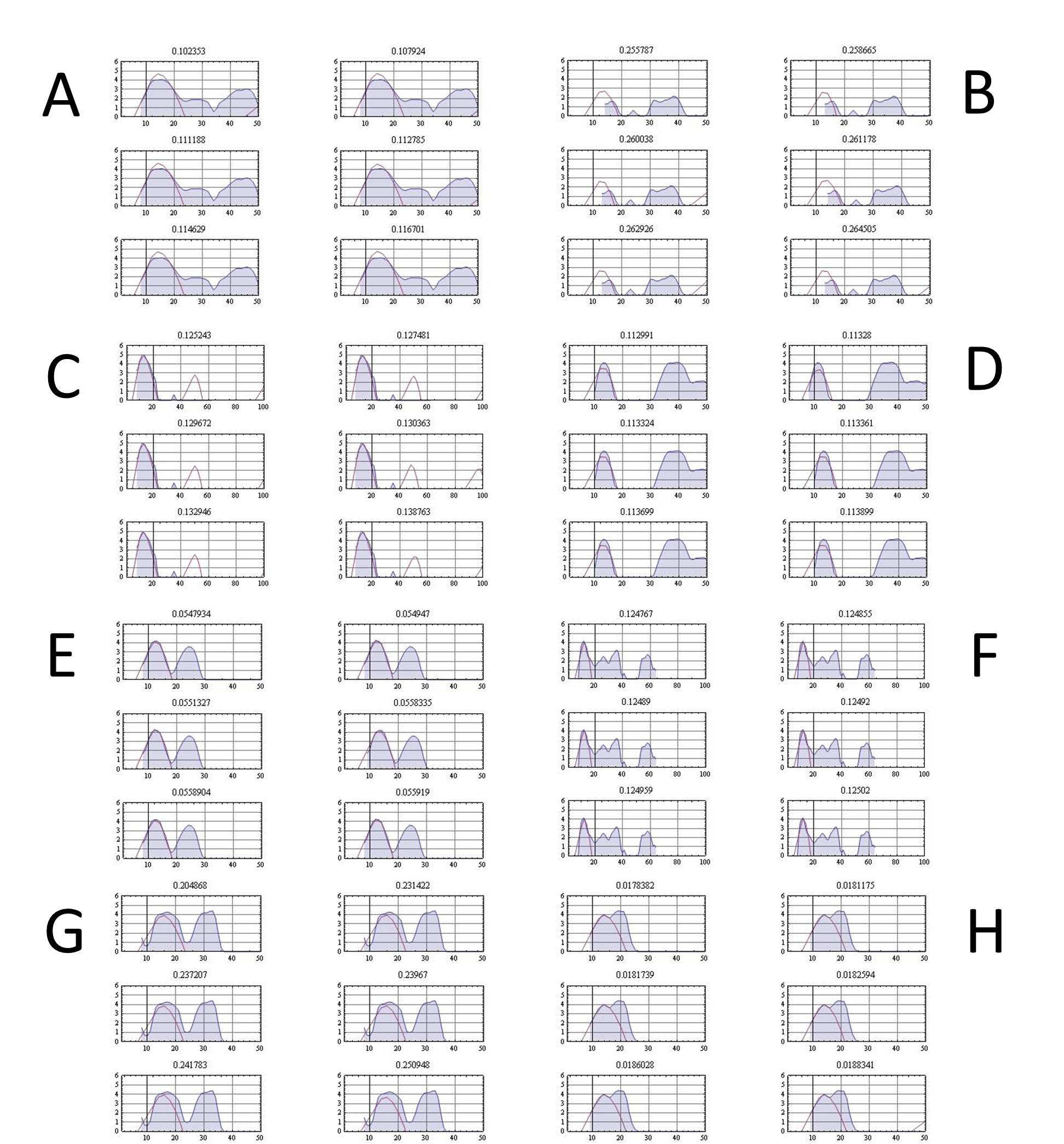

Supplement: Figure S4 — Graphic representations of the six best fits to the first wave of parasitemia for datasets 63, 64, 67, 69, 70, 71, 73 and 74 (A–H). X axes are days, y axes are decadic logarithms of parasite density. The numbers above the graphs are the errors calculated using equation (6). (TIF) [file pone.0034040.s004.tif]

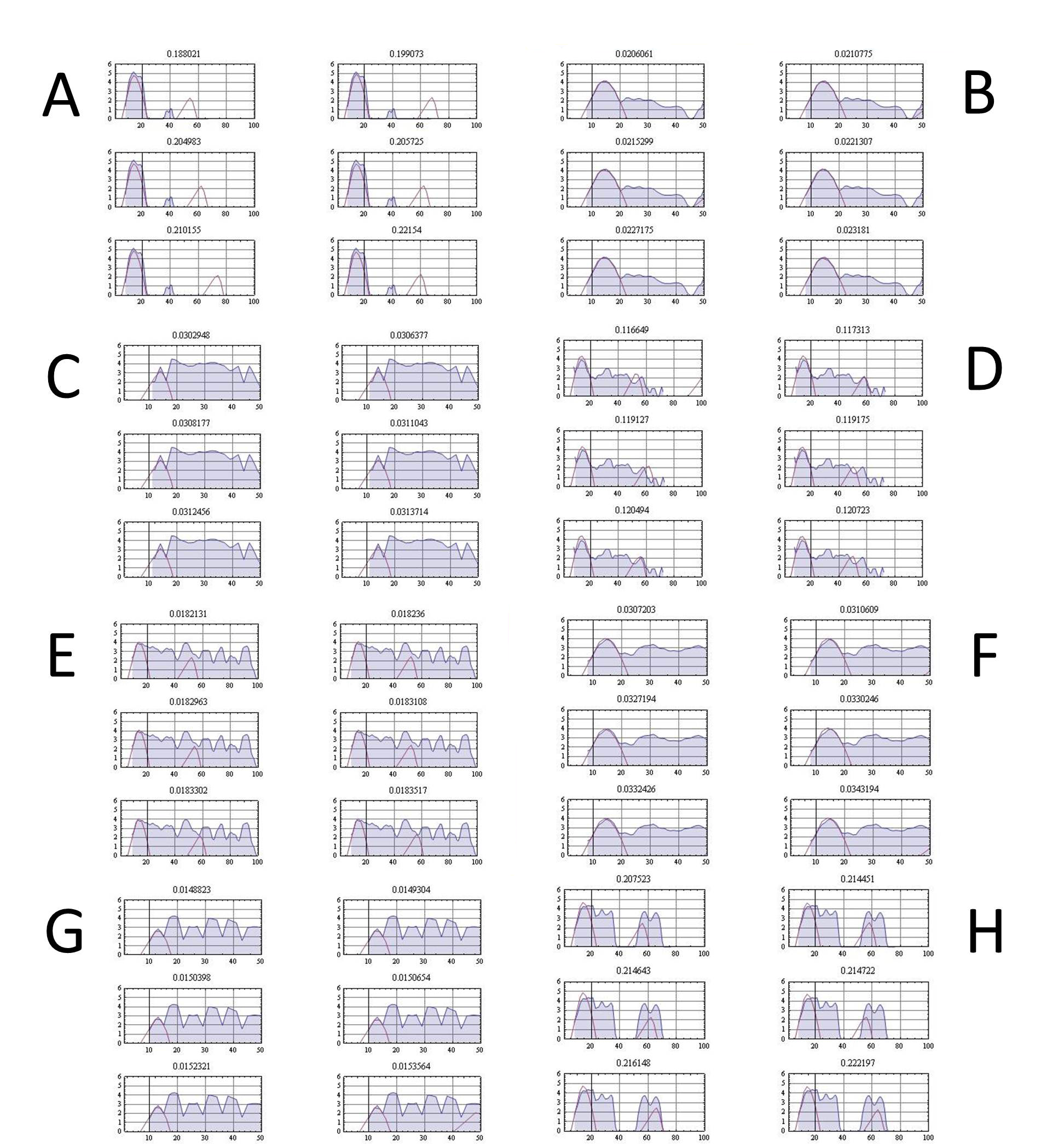

Supplement: Figure S5 — Graphic representations of the six best fits to the first wave of parasitemia for datasets 76, 77, 78, 79, 80, 81, 82 and 83 (A–H). X axes are days, y axes are decadic logarithms of parasite density. The numbers above the graphs are the errors calculated using equation (6). (TIF) [file pone.0034040.s005.tif]

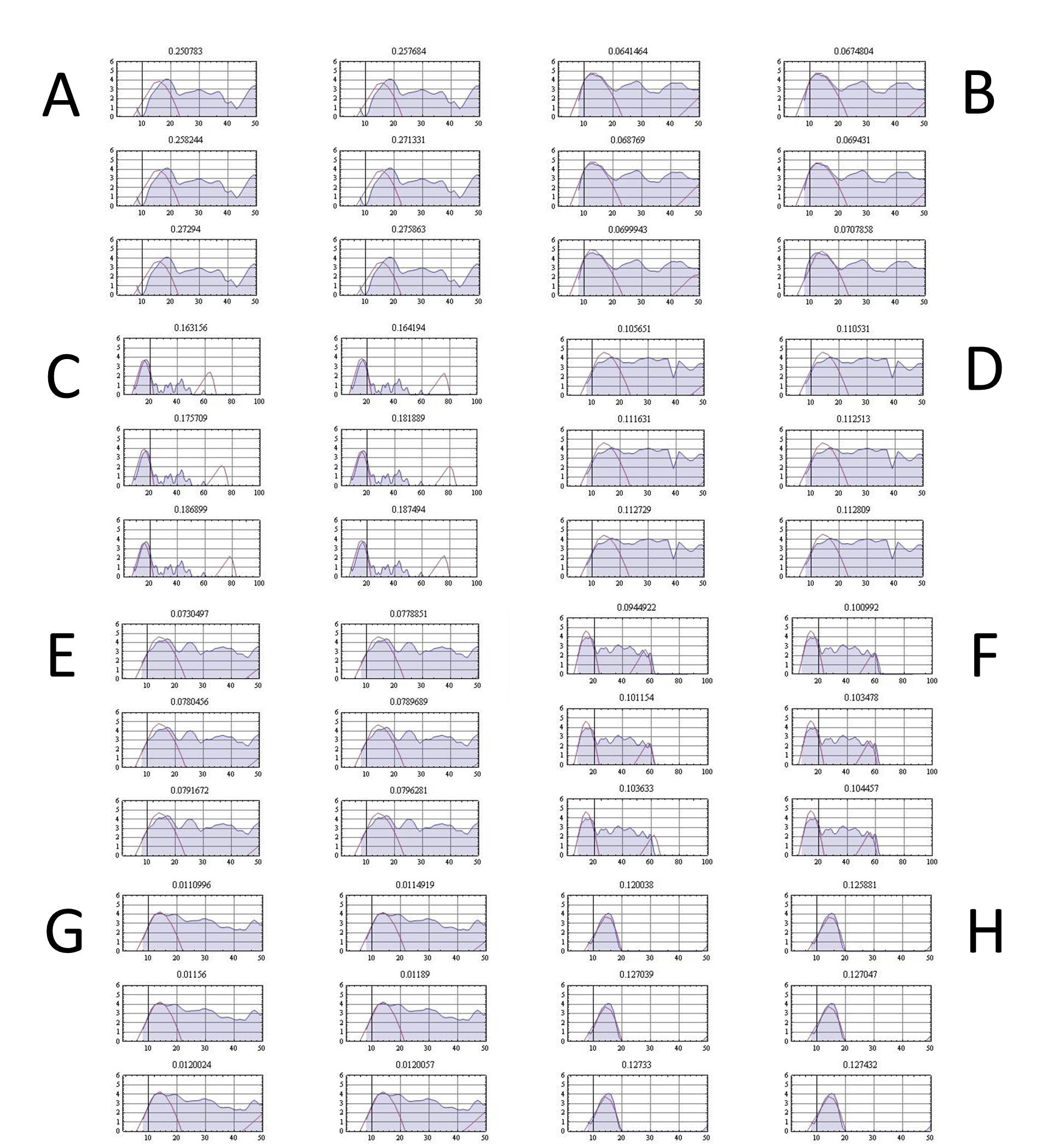

Supplement: Figure S6 — Graphic representations of the six best fits to the first wave of parasitemia for datasets 84, 85, 86, 87, 88, 89, 90 and 91 (A–H). X axes are days, y axes are decadic logarithms of parasite density. The numbers above the graphs are the errors calculated using equation (6). (TIF) [file pone.0034040.s006.tif]

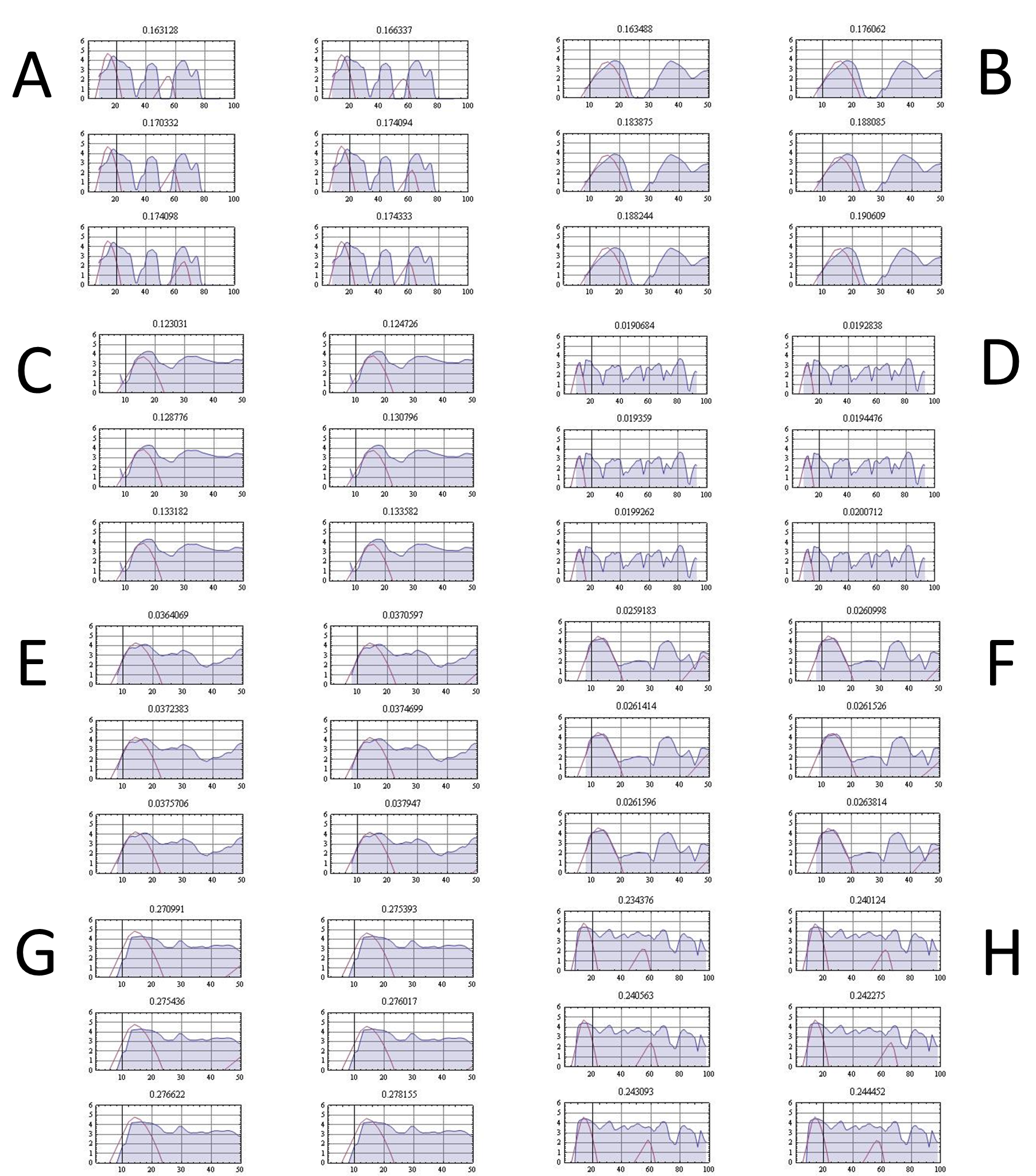

Supplement: Figure S7 — Graphic representations of the six best fits to the first wave of parasitemia for datasets 92, 93, 94, 95, 96, 97, 98 and 99 (A–H). X axes are days, y axes are decadic logarithms of parasite density. The numbers above the graphs are the errors calculated using equation (6). (TIF) [file pone.0034040.s007.tif]

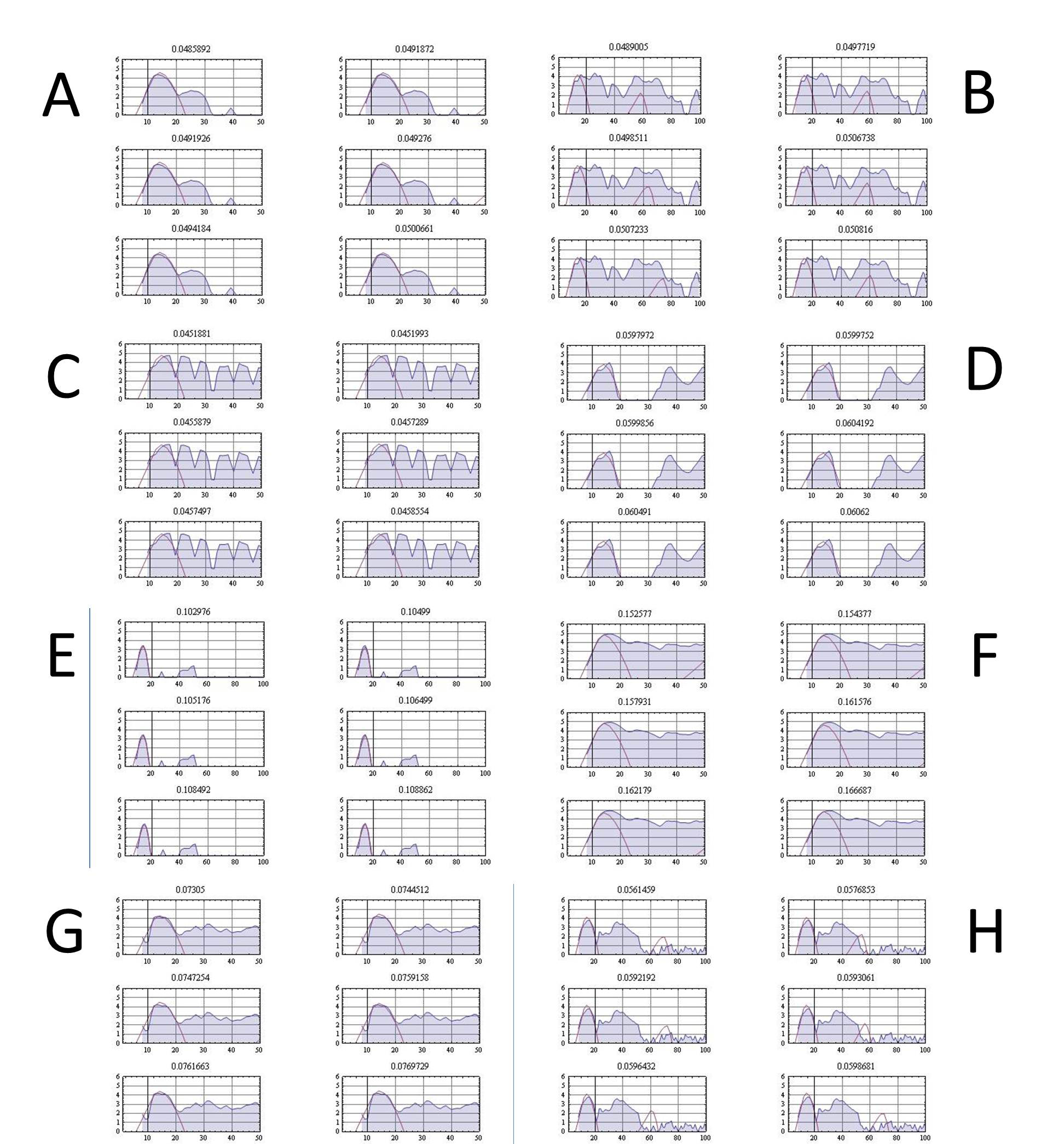

Supplement: Figure S8 — Graphic representations of the six best fits to the first wave of parasitemia for datasets 100, 101, 102, 103, 105, 106, 107 and 109 (A–H). X axes are days, y axes are decadic logarithms of parasite density. (TIF) [file pone.0034040.s008.tif]

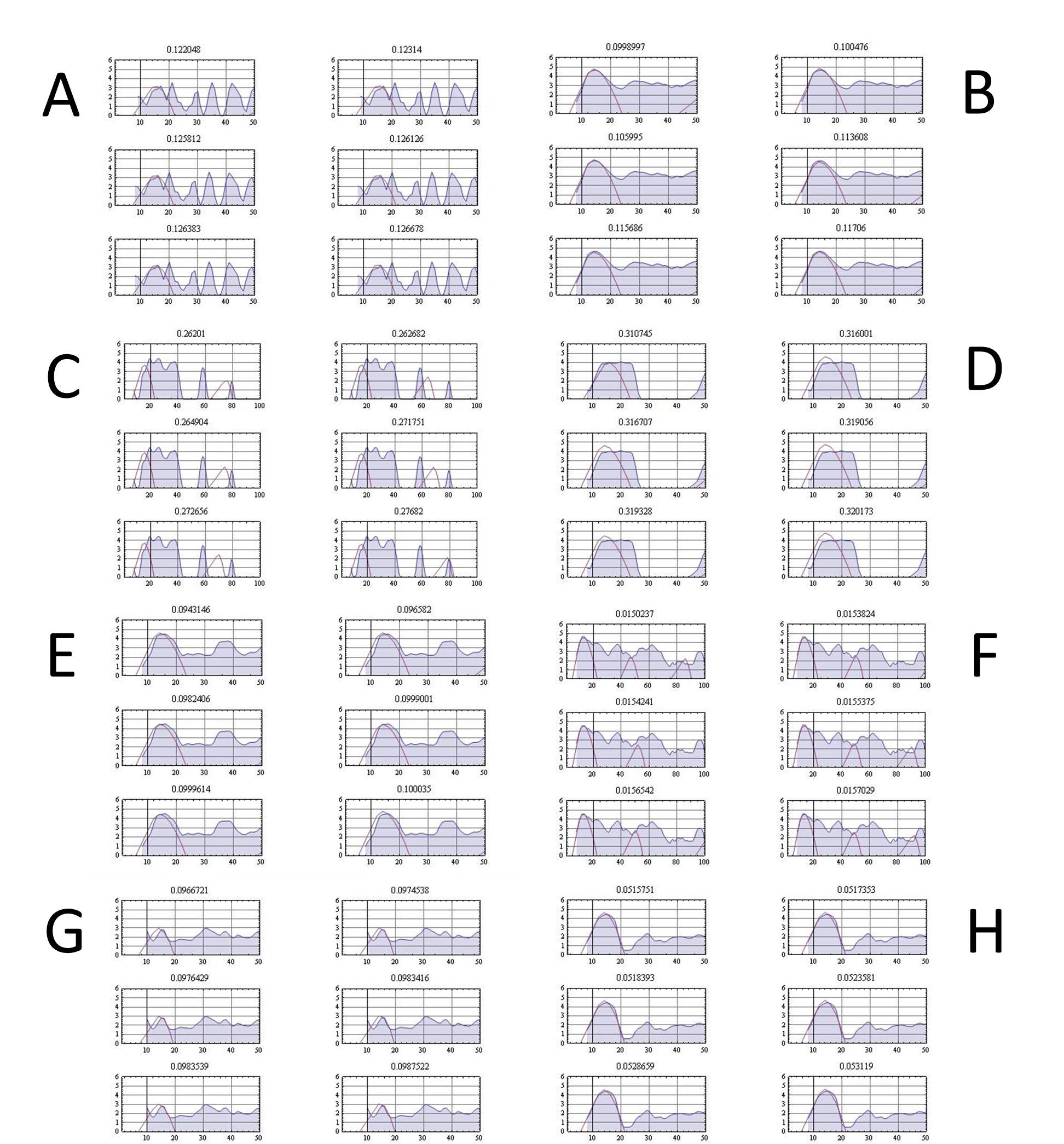

Supplement: Figure S9 — Graphic representations of the six best fits to the first wave of parasitemia for datasets 110, 111, 114, 115, 116, 117, 118 and 119 (A–H). X axes are days, y axes are decadic logarithms of parasite density. The numbers above the graphs are the errors calculated using equation (6). (TIF) [file pone.0034040.s009.tif]

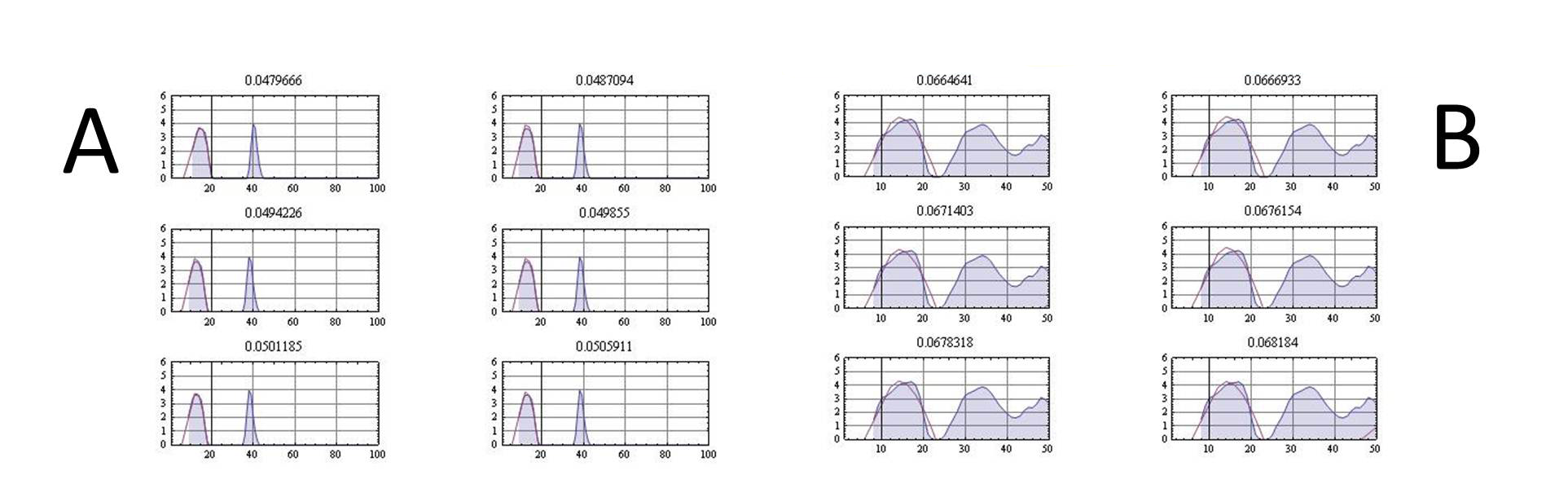

Supplement: Figure S10 — Graphic representations of the six best fits to the first wave of parasitemia for datasets 120 and 121 (A–B). X axes are days, y axes are decadic logarithms of parasite density. The numbers above the graphs are the errors calculated using equation (6). (TIF) [file pone.0034040.s010.tif]

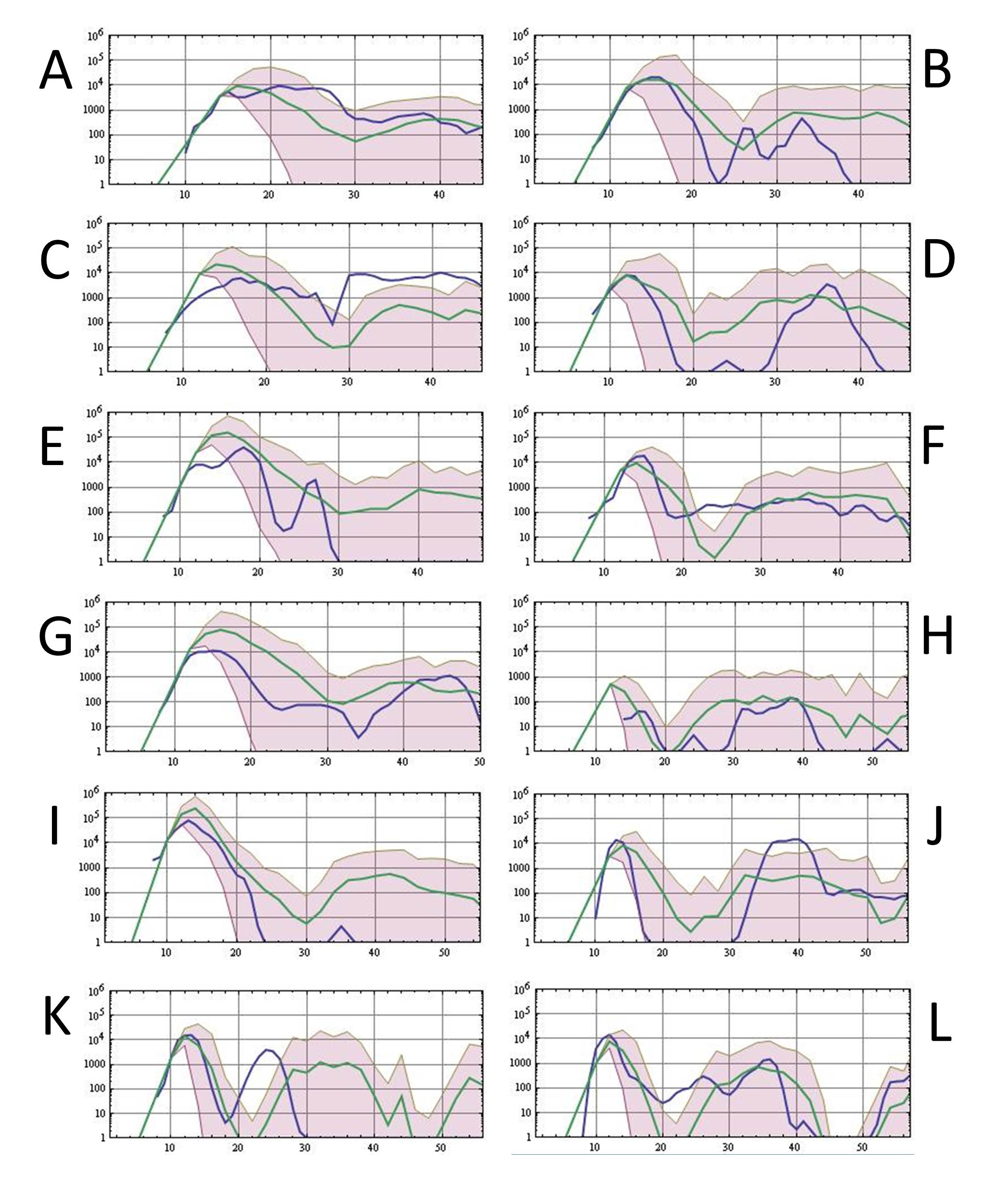

Supplement: Figure S11 — Best ensemble fits to the entire course of infection for data sets 37, 39, 40, 41, 44, 45, 46, 48, 50, 51, 52 and 54 (A–L). Blue lines are the MT data, green lines are the ensemble means and shaded purple areas are ensemble envelopes. X axes are days, y axes are decadic logarithms of parasite density. (JPG) [file pone.0034040.s011.jpg]

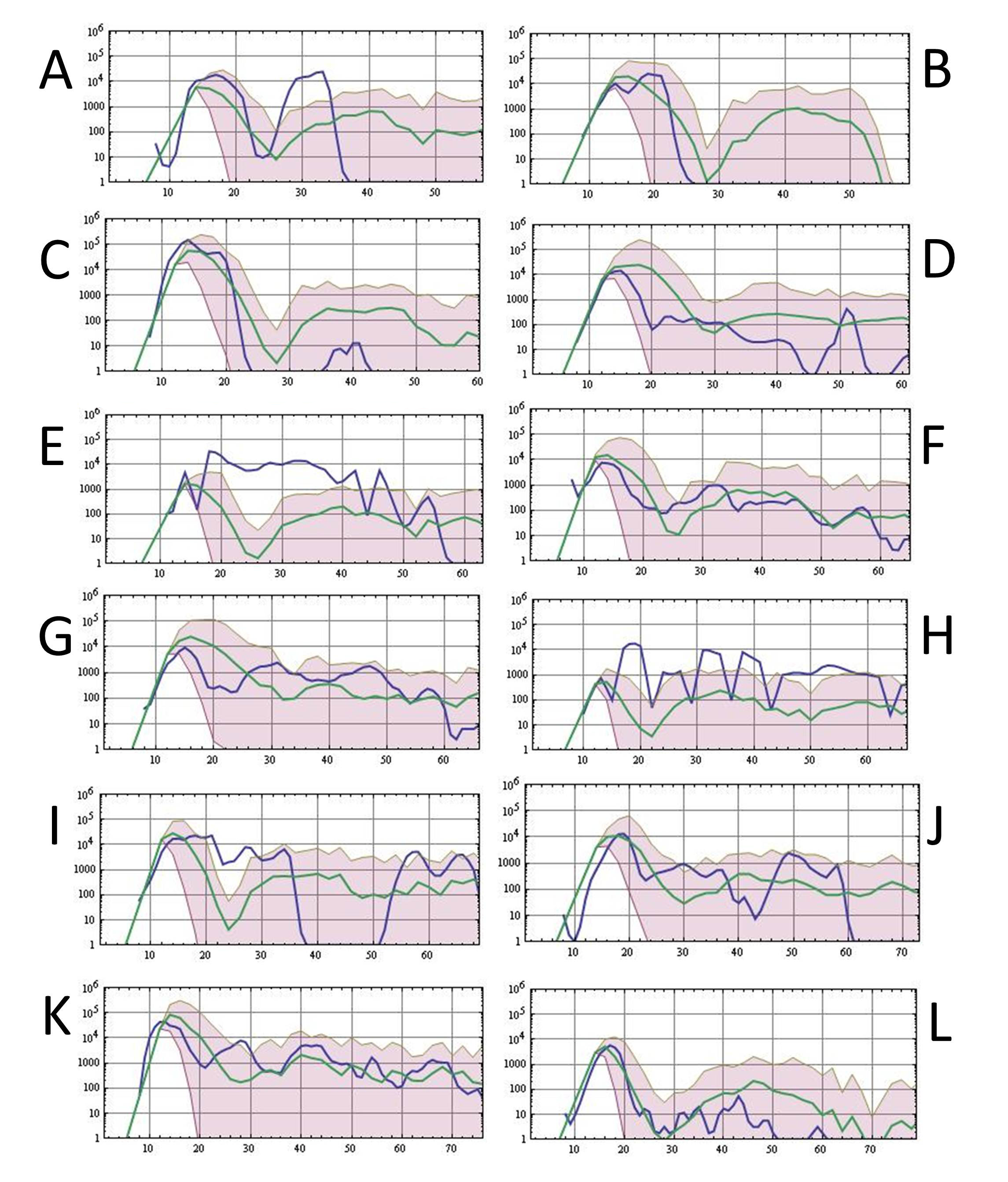

Supplement: Figure S12 — Best ensemble fits to the entire course of infection for data sets 55, 56, 57, 58, 59, 60, 62, 63, 64, 67, 69 and 70 (A–L). Blue lines are the MT data, green lines are the ensemble means and shaded purple areas are ensemble envelopes. X axes are days, y axes are decadic logarithms of parasite density. (TIF) [file pone.0034040.s012.tif]

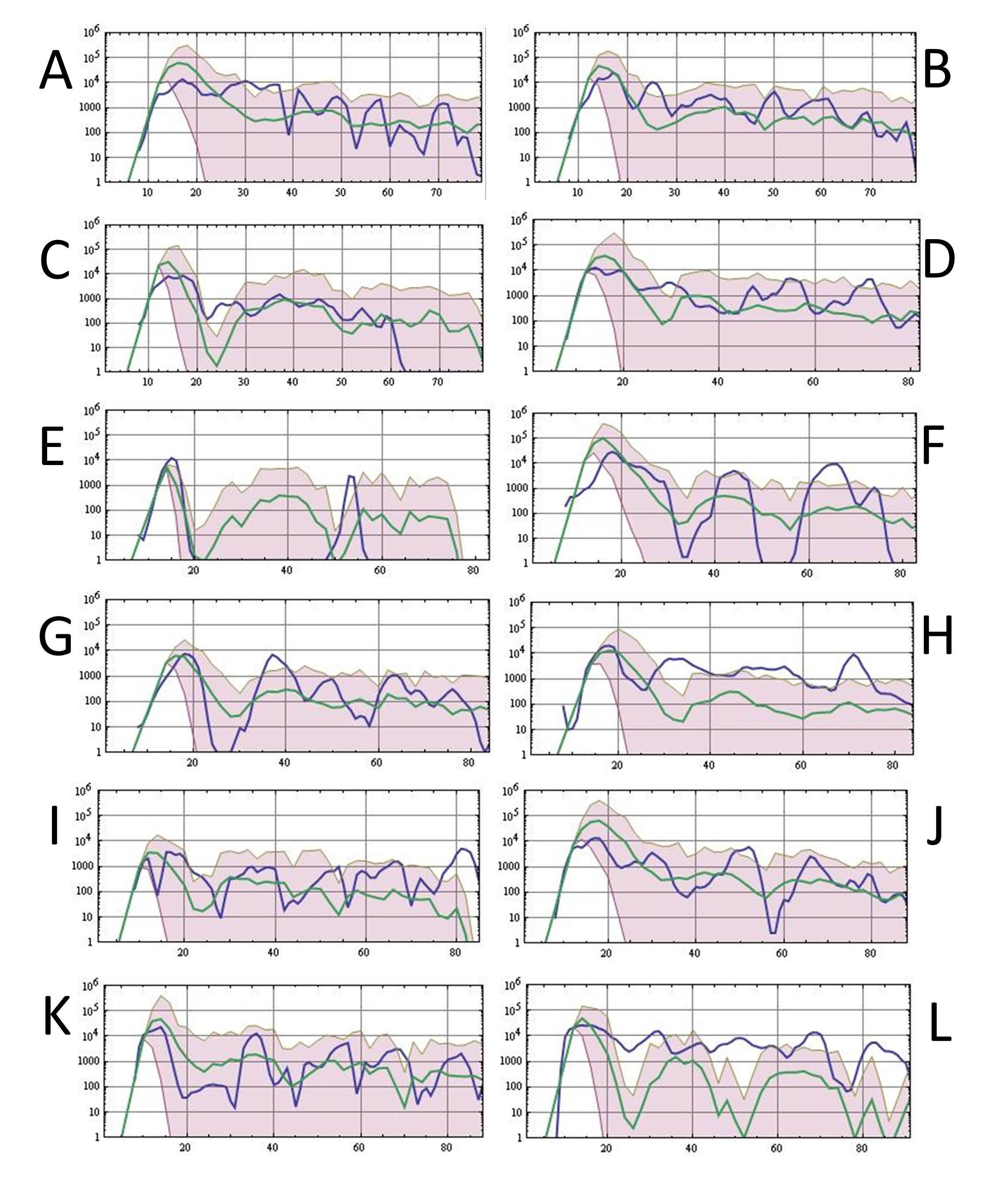

Supplement: Figure S13 — Best ensemble fits to the entire course of infection for data sets 71, 73, 74, 76, 77, 78, 79, 80, 81, 82, 83 and 84 (A–L). Blue lines are the MT data, green lines are the ensemble means and shaded purple areas are ensemble envelopes. X axes are days, y axes are decadic logarithms of parasite density. (TIF) [file pone.0034040.s013.tif]

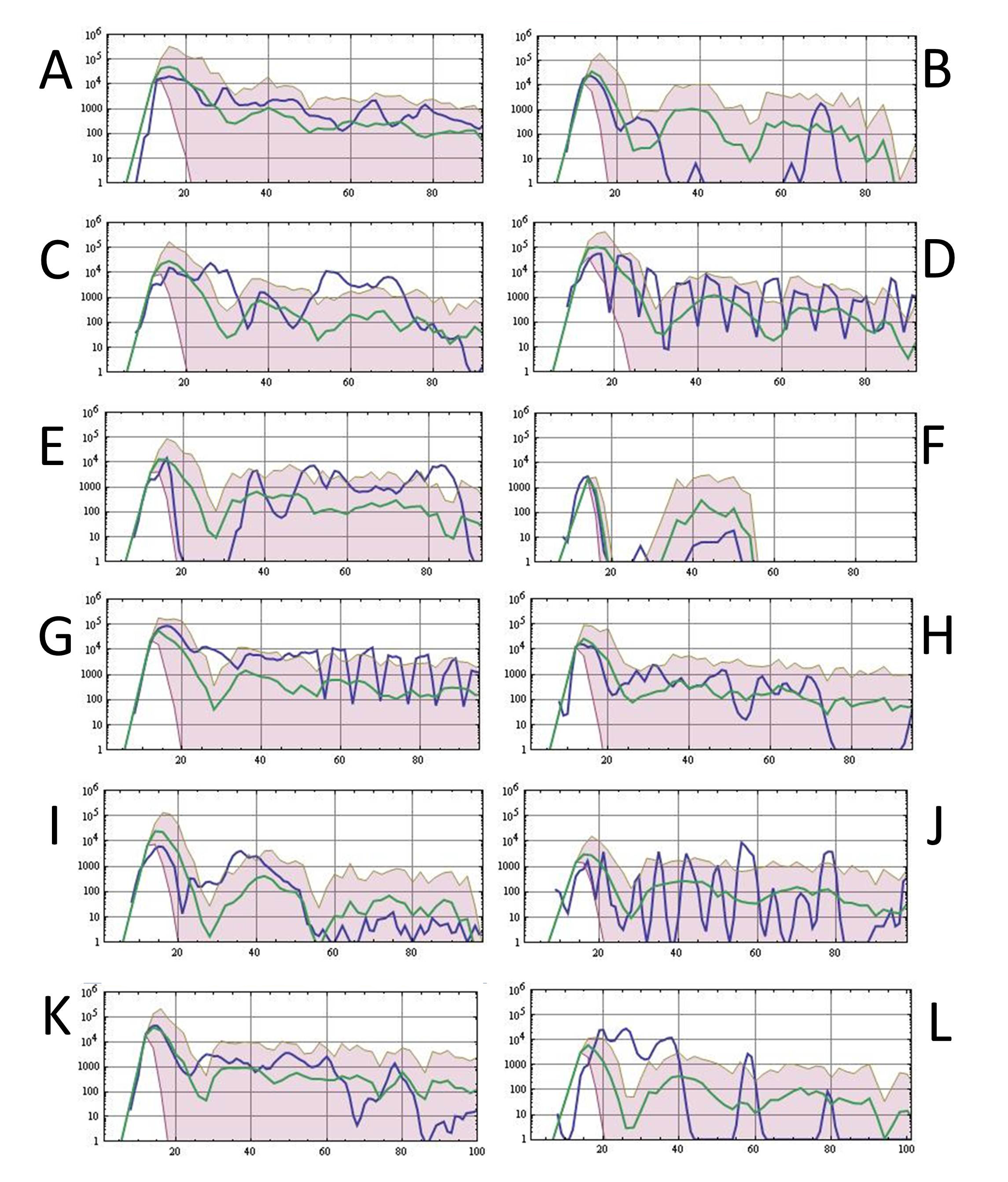

Supplement: Figure S14 — Best ensemble fits to the entire course of infection for data sets 85, 86, 87, 88, 89, 90, 91, 92, 93, 94, 95 and 96 (A–L). Blue lines are the MT data, green lines are the ensemble means and shaded purple areas are ensemble envelopes. X axes are days, y axes are decadic logarithms of parasite density. (TIF) [file pone.0034040.s014.tif]

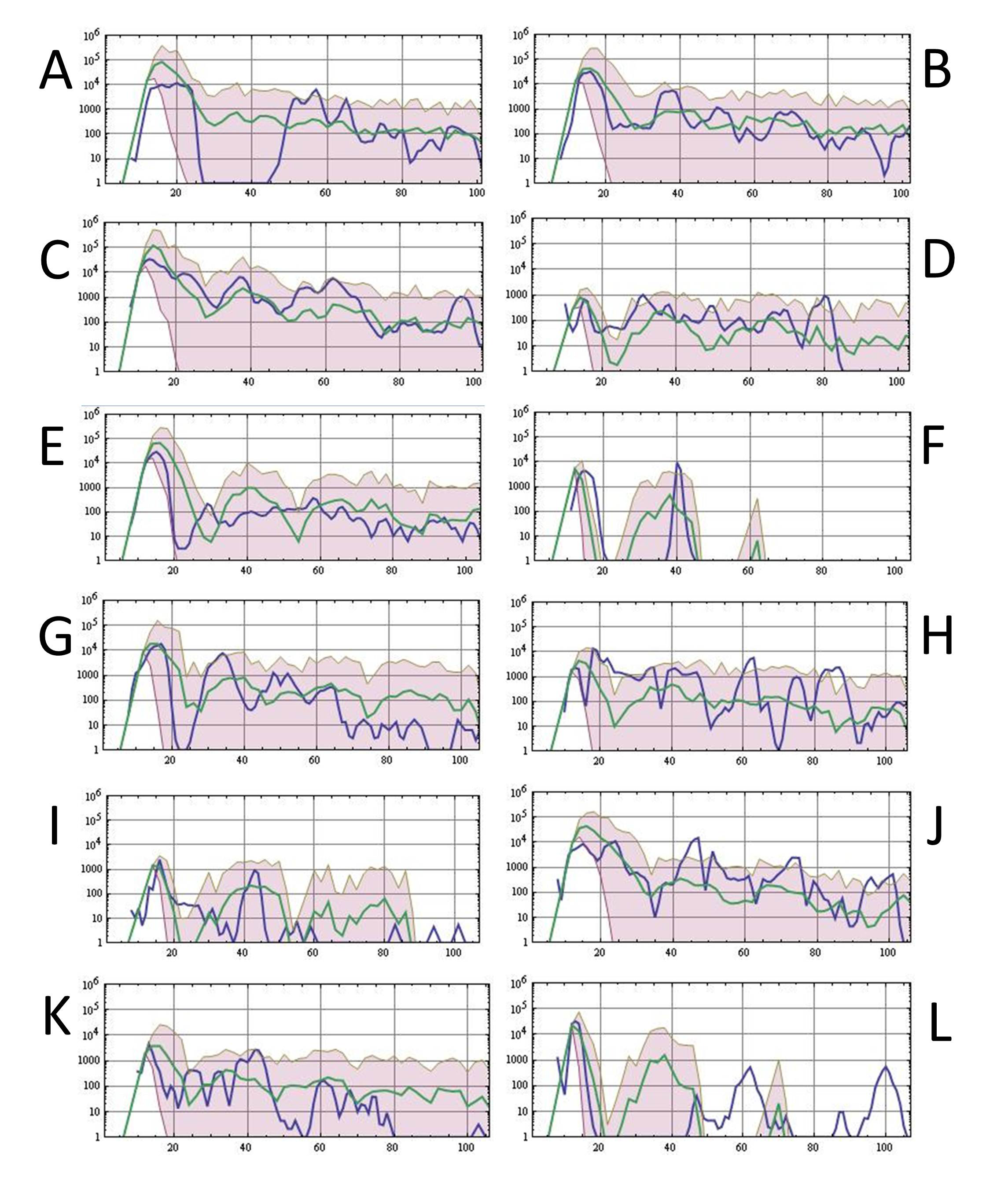

Supplement: Figure S15 — Best ensemble fits to the entire course of infection for data sets 97, 98, 99, 100, 101, 102, 103, 105, 106, 107, 109 and 110 (A–L). Blue lines are the MT data, green lines are the ensemble means and shaded purple areas are ensemble envelopes. X axes are days, y axes are decadic logarithms of parasite density. (TIF) [file pone.0034040.s015.tif]

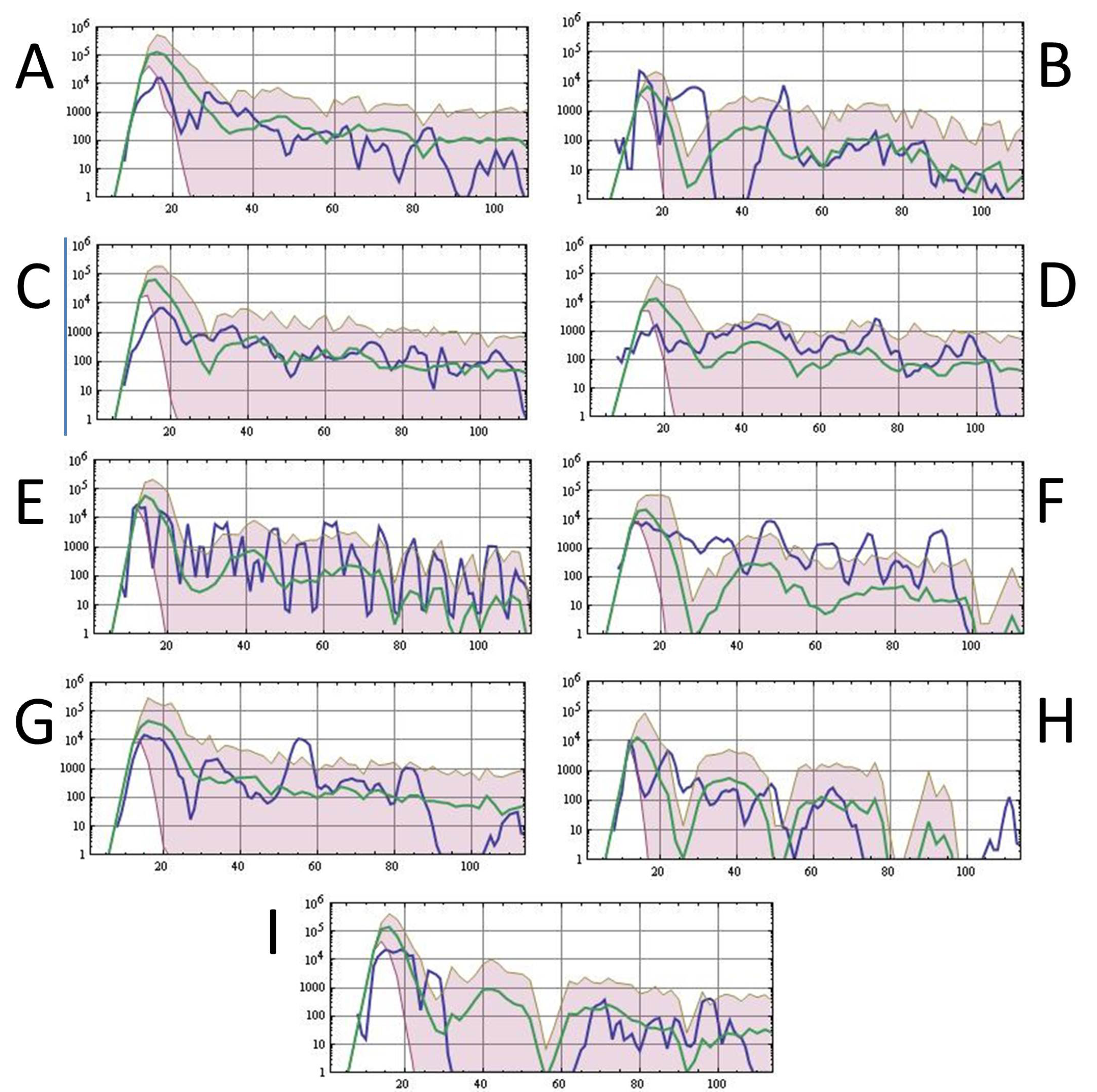

Supplement: Figure S16 — Best ensemble fits to the entire course of infection for data sets 111, 114, 115, 116, 117, 118, 119, 120 and 121 (A–I). Blue lines are the MT data, green lines are the ensemble means and shaded purple areas are ensemble envelopes. X axes are days, y axes are decadic logarithms of parasite density. (TIF) [file pone.0034040.s016.tif]

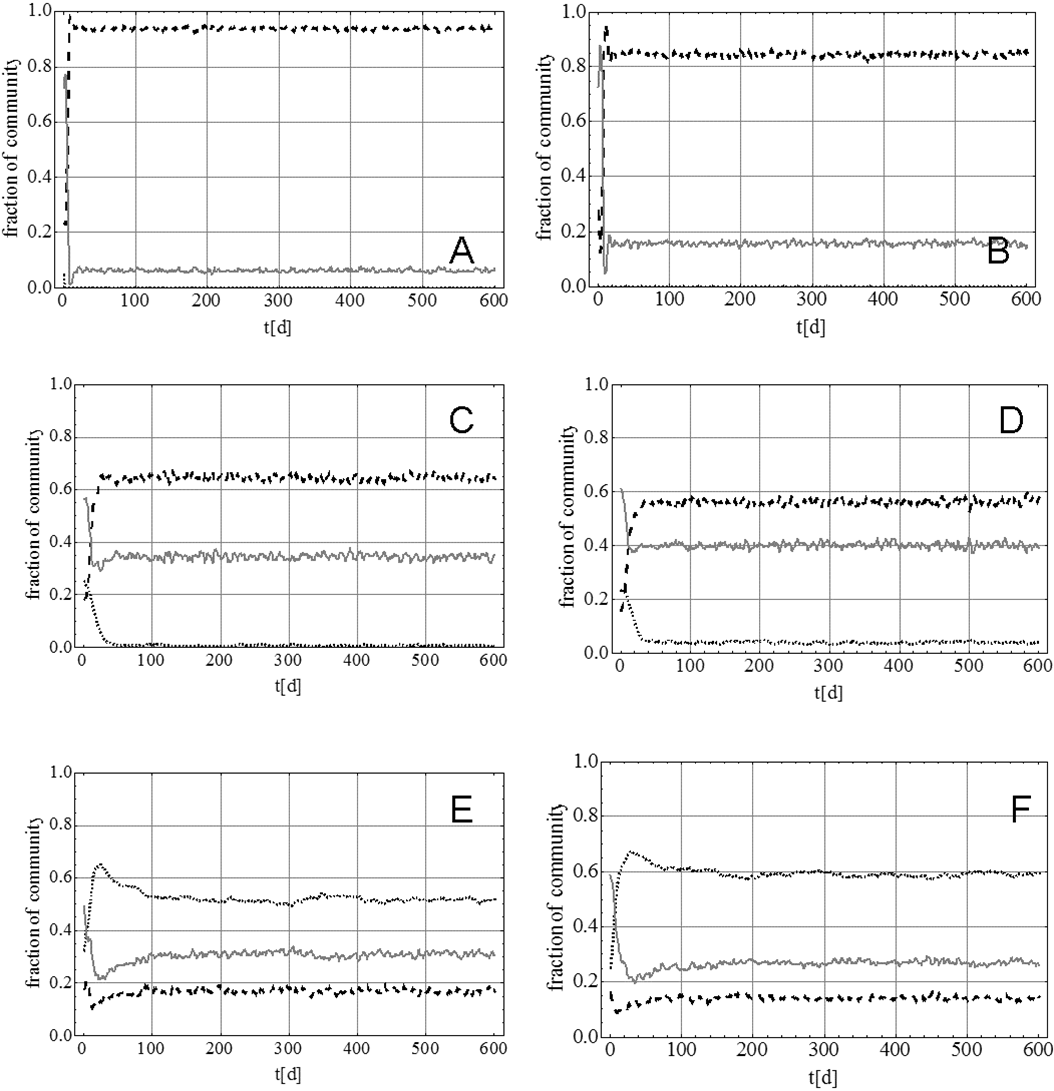

Supplement: Figure S17 — 6 panels comparing random versus best parameter based community predictions of the model. The panels on the left hand side are community runs using random parameters. The panels on the right hand side are community runs using parameters from the model calibration. Community size is n = 2000. Panels A and B compare the community prevalences at an EIR of 1 per parasite reproductive cycle (182.5 per annum), Panels C and D compare the community prevalences at an EIR of 0.1 per cycle (18.3 per annum), and panels E and F compare community prevalences at an EIR of 0.01 per cycle (1.83 per annum). The dotted black lines denote fraction of uninfected RBC, the dashed black denotes iRBC, and the solid gray denotes infected but below limit of detection by light microscopy (10 parasites per microliter). (TIF) [file pone.0034040.s017.tif]
