# Supplementary material for: Mathematical Modeling of Malaria Infection with Innate and Adaptive Immunity in Individuals and Agent-Based Communities
Source: PLoS One. 2012 Mar 28;7(3):e34040. doi: 10.1371/journal.pone.0034040 (PMC3314696; doi:10.1371/journal.pone.0034040)
Supplement: Table S1 — Description of all variables, parameters and indices used in the model. (DOC) [file pone.0034040.s018.doc]

| **Variables** | **Definition** | **Unit** |
| --- | --- | --- |
| *x(t)* | Concentration of uninfected cells at time point t | μL^-1^ |
| *y(t)* | Concentration of infected cells at time point t | μL^-1^ |
| *a(t)* | Strength of innate immune response at time point t | arbitrary |
| *b(t)* | Strength of adaptive immune response at time point t | arbitrary |
| *q_0_(t)* | Fraction to which adaptive immunity decreases at time point t due to AV |  |
| ***Parameters*** |  |  |
| *A* | Parasite density above which an innate immune response is triggered | μL^-1^ |
| *B* | Parasite density above which an adaptive immune response is triggered | μL^-1^ |
| *p* | Probability of a merozoite to invade an uninfected red blood cell |  |
| *r* | parasite reproduction rate |  |
| *f_a_* | efficiency of innate, nonspecific immunity to clear parasites |  |
| *f_b_* | efficiency of adaptive, specific immunity to clear parasites |  |
| *m* | number of antigenically distinct variant clusters |  |
| *δ* | decay rate | per 48h |
| *x_0_* | normal concentration of uninfected red blood cells | μL^-1^ |
| *y_C_* | cut off parastite density below which infection is presumed cleared | μL^-1^ |
| *M* | number of Merozoites (result of r*y(t)) | μL^-1^ |
| *u* | range for modification of e during calibration step 2 |  |
| *v* | range for modification of ε during calibration step 2 |  |
| ***Indices*** |  |  |
| *MT* | Malaria therapy |  |
| *M* | Maximum |  |
| *C* | Cut off |  |
| *0* | initial/normal |  |
| *a* | concerning innate immunity |  |
| *b* | concerning adaptive immunity |  |
