# Supplementary material for: Mathematical Modeling of Malaria Infection with Innate and Adaptive Immunity in Individuals and Agent-Based Communities
Source: PLoS One. 2012 Mar 28;7(3):e34040. doi: 10.1371/journal.pone.0034040 (PMC3314696; doi:10.1371/journal.pone.0034040)
Supplement: Table S2 — Overview over the MT data used in the present study and allocation of numbers to each set of MT data. The columns labeled ‘#’ are the numbers assigned to each dataset over the course of this study to facilitate data processing. (DOC) [file pone.0034040.s019.doc]

| # | MT host | # | MT host | # | MT host | # | MT host | # | MT host |
| --- | --- | --- | --- | --- | --- | --- | --- | --- | --- |
| 1 | S-954 | 26 | S-501 | 52 | S-410 | 79 | S-1246 | 105 | S-541 |
| 2 | S-991 | 27 | S-1302 | 53 | S-811 | 80 | S-607 | 106 | S-544 |
| 3 | S-1003 | 28 | S-1317 | 54 | S-1088 | 81 | S-558 | 107 | S-556 |
| 4 | S-941 | 29 | G-54 | 55 | S-775 | 82 | S-519 | 108 | S-1326 |
| 5 | S-965 | 30 | S-909 | 56 | G-289 | 83 | S-759 | 109 | S-535 |
| 6 | S-966 | 31 | S-1299 | 57 | G-201 | 84 | S-566 | 110 | S-697 |
| 7 | S-604 | 32 | S-1332 | 58 | S-457 | 85 | G-156 | 111 | S-1102 |
| 8 | S-789 | 33 | S-760 | 59 | S-1329 | 86 | S-606 | 112 | S-785 |
| 9 | S-700 | 34 | S-824 | 60 | S-815 | 87 | S-741 | 113 | S-1337 |
| 10 | S-1272 | 35 | S-986 | 61 | S-1291 | 88 | S-859 | 114 | S-392 |
| 11 | S-884 | 36 | S-1285 | 62 | S-548 | 89 | S-994 | 115 | S-1114 |
| 12 | S-1216 | 37 | S-1323 | 63 | G-300 | 90 | G-380 | 116 | S-953 |
| 13 | S-1313 | 38 | S-444 | 64 | S-1243 | 91 | S-200 | 117 | G-246 |
| 14 | S-707 | 39 | G-122 | 65 | S-1338 | 92 | S-895 | 118 | S-858 |
| 15 | S-940 | 40 | S-622 | 67 | S-1295 | 93 | G-243 | 119 | G-228 |
| 16 | S-1220 | 41 | S-851 | 68 | S-561 | 94 | S-514 | 120 | S-439 |
| 16 | S-1220 | 42 | S-1263 | 69 | S-793 | 95 | S-465 | 121 | S-713 |
| 17 | S-787 | 43 | S-1232 | 70 | G-452 | 96 | S-1301 | 125 | G-157 |
| 18 | S-910 | 44 | S-1296 | 71 | S-1249 | 97 | S-1342 | 146 | S-406 |
| 19 | S-972 | 45 | S-402 | 72 | S-774 | 98 | S-463 | 179 | G-251 |
| 20 | S-505 | 46 | S-1336 | 73 | S-807 | 99 | S-731 | 182 | G-104 |
| 21 | S-660 | 47 | S-475 | 74 | S-988 | 100 | S-937 | 200 | G-159 |
| 22 | S-882 | 48 | G-103 | 75 | S-551 | 101 | S-1321 | 201 | G-212 |
| 23 | S-1018 | 49 | S-1242 | 76 | S-782 | 102 | S-995 |  |  |
| 24 | S-565 | 50 | S-1312 | 77 | S-1221 | 103 | S-502 |  |  |
| 25 | S-732 | 51 | S-1298 | 78 | S-1297 | 104 | S-678 |  |  |
