# Supplementary material for: Mathematical Modeling of Malaria Infection with Innate and Adaptive Immunity in Individuals and Agent-Based Communities
Source: PLoS One. 2012 Mar 28;7(3):e34040. doi: 10.1371/journal.pone.0034040 (PMC3314696; doi:10.1371/journal.pone.0034040)
Supplement: Table S3 — Comparative statistics between the output of our model, the MT data we used for calibration and data presented by Gatton et al 2006 [13] . (DOC) [file pone.0034040.s020.doc]

| Statistic | Our Model (n=200) | MT dataset (n=125) | t-test | Gatton, 2006;  McLendon (n=56) | Gatton 2006;  El Limon/S. Cooper (n=34) |
| --- | --- | --- | --- | --- | --- |
| Parasitemia at 1st maximum | 4.18 (2.50-5.01) | 4.16 (1.61-5.26) | ns | 4.49(3.93-4.94) | 4.81(4.47-5.31) |
| Number of local maxima | 5 (1-19) | 7 (1-35) | ns | 6.5(1-24) | 10 (1-27) |
| Geometric mean Intervals between maxima | 13.02(3-92) | 13.3 (2-202) | ns | 20.5(10-32) | 22(14.2-29.3) |
| Length of Infection | 56(9-134) | 48 (4-205) | ns | 117(12-590) | 239(10-590) |
| Percent>10 µL^-1^ in first half of infection | 48% (25%-69%) | 94%(48%-99%) | p<0.05 | 79(52-100) | 0.67(0.48-1.0) |
| Percent>10 µL^-1^ in second half of infection | 33% (0%-75%) | 90%(11%-98%) | P<0.05 | 60(37-100) | 0.61(37-1.0) |
| Slope through the first 5 maxima | -0.0326 (-0.011-0.059) | -0.022 (-0.008-0.035) | p<0.05 | -0.014(-0.005-0.031) | -0.014(-0.006-0.029) |
